# A broad-host-range inhalable phage cocktail for treatment of multidrug-resistant *Pseudomonas aeruginosa* pulmonary infections

**DOI:** 10.3389/fcimb.2026.1912918

**Published:** 2026-09-15

**Authors:** Zixun Lin, Xiaofu Wan, Xiangke Duan, Shuang Liang, Xiaoqian Wang, Jiuxin Qu, Chuandong Zhou, Min Ming, Yukun Zeng, Shuhong Han, Yijia Ling, Wenheng Gan, Ke Wang, Liang Yang, Xin Zhou, Hongzhou Lu, Jiayin Shen

**Affiliations:** 1 National Clinical Research Centre for Infectious Diseases, The Third People’s Hospital of Shenzhen and The Second Affiliated Hospital of Southern University of Science and Technology, Southern University of Science and Technology, Shenzhen, China; 2 School of Medicine, Southern University of Science and Technology, Shenzhen, China; 3 PhageNovoTech Co., Ltd., Shenzhen, China; 4 College of Veterinary Medicine, Institute of Comparative Medicine, Yangzhou University, Yangzhou, China; 5 Department of Pulmonary and Critical Care Medicine, The First Affiliated Hospital of Guangxi Medical University, Nanning, China

**Keywords:** bacteriophage therapy, broad-host-range phage, inhalation delivery, multidrug-resistant *Pseudomonas aeruginosa*, phage cocktail

## Abstract

**Introduction:**

The growing threat of multidrug-resistant (MDR) *Pseudomonas aeruginosa* (*P. aeruginosa)* has created an urgent need for novel therapeutic strategies to combat refractory lung infections. The limited efficacy of traditional antibiotics against this pathogen highlights the necessity of developing alternative treatments. Our study aims to develop a broad-spectrum bacteriophage cocktail suitable for nebulization and to evaluate its antibacterial efficacy and safety in respiratory infections caused by MDR *P. aeruginosa*.

**Methods:**

We constructed a seven-phage cocktail by selecting phages with complementary host ranges, strong lytic activity, and different receptor targets. The antimicrobial and anti-biofilm activity of the cocktail was assessed *in vitro* against clinical MDR *P. aeruginosa* isolates. The therapeutic efficacy and safety were further investigated in a murine pneumonia model following nebulized administration.

**Results:**

The phage cocktail demonstrated lytic activity against nearly 90% of 152 clinical *P. aeruginosa i*solates, indicating broad coverage. Compared to single phages or antibiotics, the cocktail showed superior activity in both inhibiting and disrupting biofilms. In the murine pneumonia model, nebulized phage therapy improved survival, reduced bacterial burdens in lung and liver, and alleviated infection-related histopathological damage. Safety assessment revealed no obvious hematological, biochemical or histopathological abnormalities in both acute and long-term administration.

**Conclusion:**

High-dose nebulized administration of this phage cocktail reduced bacterial burden and showed favorable tolerability in a murine model of MDR *P. aeruginosa* pneumonia. These findings provide preclinical evidence supporting further evaluation of inhaled phage cocktails as a potential therapeutic strategy for refractory respiratory infections.

## Graphical Abstract

**Figure:**
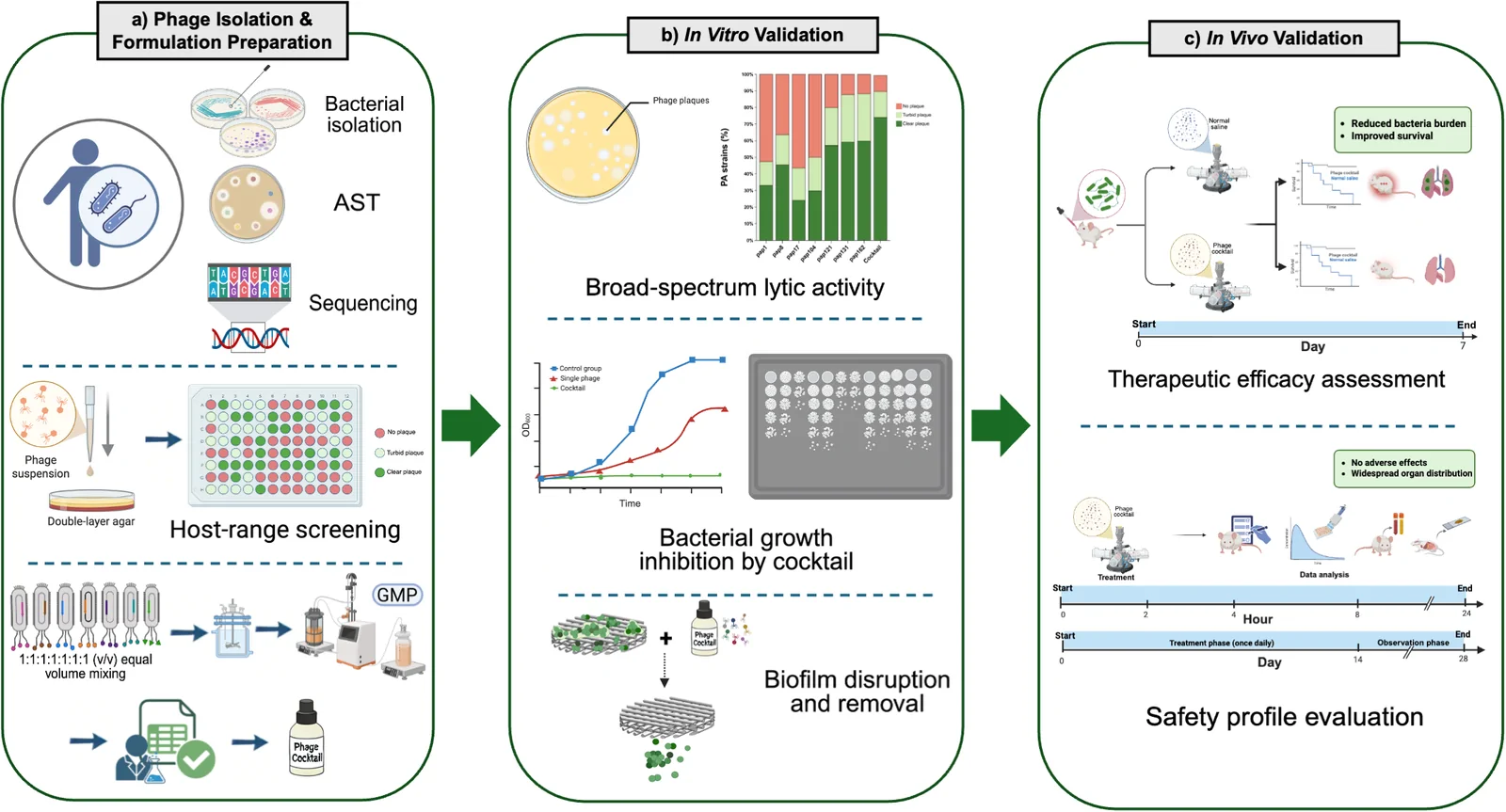

## 1 Introduction

Antimicrobial resistance (AMR) is now widely regarded as a significant challenge for healthcare systems worldwide. It leads to an increase in global mortality rates and imposes a heavy burden on the global health system. The increasing prevalence of extensively drug-resistant (XDR) and MDR bacteria have substantially contributed to the global burden of antimicrobial resistance. In some regions, the carbapenem-resistant rates of certain pathogens have exceeded 30%. Among these pathogens, carbapenem-resistant *Pseudomonas aeruginosa* (CRPA) is particularly concerning (Ma et al., 2023).

The emergence of MDR *P. aeruginosa* poses a severe threat to public health (Kunz Coyne et al., 2022). This pathogen has a notable capacity for rapid genetic adaptation (Mulcahy et al., 2010), which enables it to develop strong antibiotic resistance. MDR *P. aeruginosa* is a common cause of respiratory infections, including bronchiectasis, cystic fibrosis (CF) (Malhotra et al., 2019), chronic obstructive pulmonary disease (COPD) (Lusuardi et al., 2003), and pneumonia (Gu and Pang, 2025), and is often associated with increased morbidity and mortality. One of the main factors contributing to its persistence is the ability of *P. aeruginosa* to form biofilms, particularly in the lungs of patients with CF (Ciofu and Tolker-Nielsen, 2015). In these biofilms, bacteria can evade host immune responses and antimicrobial treatments, thereby facilitating long-term colonization and leading to persistent infections (Jennings et al., 2021). This underscores the need for alternative antimicrobial approaches targeting respiratory tract infections caused by *P. aeruginosa*.

With the increasing threat posed by AMR to the global medical system, it is becoming more important to develop alternative treatment strategies. In recent years, bacteriophage therapy has gained renewed attention as a potential option against antimicrobial-resistant bacterial pathogens. Bacteriophages are viruses that infect and lyse bacteria (Strathdee et al., 2023). They are characterized by high host specificity (Palma and Qi, 2024) and the ability to self-replicate at the site of infection (Bhargava et al., 2021). In addition, phage therapy is generally regarded as safe and is associated with minimal side effects. Exposure to phage selective pressure can drive evolutionary trade-offs in bacteria, including reduced growth fitness (Borin et al., 2021), weakened virulence (Gao et al., 2022; Zhao et al., 2026), and even restored sensitivity to traditional antibiotics (Laure and Ahn, 2022; Zou et al., 2025). In line with this therapeutic potential, the U.S. Food and Drug Administration (FDA) has designated phages as emergency Investigational New Drugs (eIND) for managing MDR bacterial infections (Palma and Qi, 2024).

In current clinical practice, most phage therapy is implemented as a personalized phage therapy tailored to a specific patient’s isolate. Phage therapy offers high specificity. However, its development and preparation often require several weeks, which may not be suitable for critically ill patients, particularly those with respiratory infections in intensive care units (Chan et al., 2025; Kim et al., 2024). Therefore, the development of pre-optimized, broad-host-range phage cocktails is highly advantageous, particularly in emergency situations where timely administration is essential (Kim et al., 2024). Phage cocktails can delay or prevent the emergence of phage-resistant bacterial mutants, which are commonly observed in single-phage therapies.

However, the *in vivo* behavior and therapeutic potential of bacteriophage cocktails remain incompletely understood, particularly in the context of respiratory infections. In this study, we developed and evaluated a bacteriophage cocktail targeting MDR *P. aeruginosa* for pulmonary infection. Lytic phages with activity against clinically relevant *P. aeruginosa* strains were selected and assembled into a synergistic formulation. The lytic spectrum, efficiency of plating, antibacterial activity, and biofilm-disrupting capacity of the selected phages and the resulting cocktail were first characterized through a series of *in vitro* assays. Subsequently, a murine model of *P. aeruginosa* lung infection was established to evaluate the therapeutic efficacy and safety of the nebulized phage cocktail. We also analyzed the biodistribution of phages following nebulized administration and evaluated the potential of this delivery strategy to reduce bacterial burden and alleviate infection-associated pathological damage. Overall, this study provides preclinical evidence supporting further investigation of nebulized phage cocktails as a potential therapeutic approach for MDR *P. aeruginosa* respiratory tract infections.

## 2 Materials and methods

### 2.1 Bacterial strains

*P. aeruginosa* strains and *Escherichia coli* (*E. coli*) strains were cultured at 37 °C in Lysogeny Broth (LB). Kanamycin was added at 50 µg/mL. For bacteriophage amplification during the preparation of phage formulations, a defined medium based on EZ Rich Defined Medium (Coolaber, Beijing, China) was used (Banerjee et al., 2024). Artificial Sputum Medium (ASM, MesGen^®^ Biotechnology) was used to culture clinical isolates to mimic human sputum microenvironment (Kirchner et al., 2012). All medium components were sterilized and stored appropriately before use.

### 2.2 Clinical bacterial isolates and data collection

A total of 152 non-duplicate clinical *P. aeruginosa* isolates were collected from hospitalized patients in Guangxi and the Third People’s Hospital of Shenzhen. Clinical data were obtained from electronic records, and all isolates were anonymized before analysis.

### 2.3 Identification of bacteriophage host receptor types

Host receptors were examined by spot assays using *P. aeruginosa* PAO1 wild-type (WT) and its isogenic mutants, including the type IV pilus-deficient strain (PAO1 Δ*pilA)*, flagellin-deficient strain (PAO1 Δ*fliC*), and lipopolysaccharide O-antigen polymerase-deficient strain (PAO1 Δ*wzy*). Bacterial cultures were grown to mid-log phase in LB broth at 37 °C, and 100 µL of each culture was spread onto 0.5% LB agar plates to form bacterial lawns. After drying, 3 µL of phage stock (~ 1×10^8^ plaque-forming units per milliliter (PFU/mL)) was spotted onto each lawn and incubated at 37 °C for 6 h. Loss of plaques or inhibition zones on mutant lawns indicated the involvement of the corresponding receptor structure.

### 2.4 Bacterial genomic analysis

Sequencing: Genomic DNA from all *P. aeruginosa* isolates was sequenced on the Illumina PE150 platform (Novogene, China). Raw sequencing data were first processed through a quality control pipeline. Adapters and low-quality bases were trimmed using Trimmomatic v0.39 (Bolger et al., 2014). To prepare for assembly, the reads were subsampled and filtered for a minimum length of 600 bp using readfq v5.

Assembly and annotation: Genome assembly was performed on the filtered reads using Shovill v1.1.0 with default settings. The resulting draft genomes were annotated using Prokka v1.14.6 (Seemann, 2014) to identify protein-coding sequences, rRNAs, tRNAs, and other genomic features. Predicted protein sequences were classified into functional categories using the Clusters of Orthologous Groups (COG) database. Circular genome maps, including GC content and gene organization, were generated using Proksee (Grant et al., 2023) visualization software.

Genomic characterization: Genome features were analyzed based on the annotated sequences. The antimicrobial resistance genes and virulence factors were predicted by aligning with the genomic sequences in the Comprehensive Antibiotic Resistance Database (CARD) and the Virulence Factor Database (VFDB), respectively. The serotype of each *P. aeruginosa* isolate was predicted by *Pseudomonas aeruginosa* Serotyper (PAst) v1.0.

Typing and phylogeny: Multilocus sequence typing (MLST) was performed on 152 *P. aeruginosa* isolates based on seven housekeeping genes (*acsA*, *aroE*, *guaA*, *mutL*, *nuoD*, *ppsA*, and *trpE*), with allele numbers and sequence types (STs) assigned using the *P. aeruginosa* MLST database (Jolley et al., 2018). Novel profiles that did not match known STs were classified as “others.” To assess phylogenetic relationships, core-genome single-nucleotide polymorphism (SNP) analysis was performed. Genome alignments were generated using Snippy v4.6.0, followed by construction of a maximum-likelihood (ML) tree with FastTree v2.1.11 using 1000 bootstrap replicates.

### 2.5 Identification and antimicrobial testing of *P. aeruginosa*

All 152 clinical isolates were confirmed as *P. aeruginosa* using the Phoenix M50 system (Phoenix M50, bioMéreux, Marcy-l’Étoile, France). Antimicrobial susceptibility testing was performed with the same platform against clinically relevant antibiotics. Antimicrobial susceptibility was interpreted according to the “Performance Standards for Antimicrobial Susceptibility Testing” issued by the Clinical and Laboratory Standards Institute (CLSI) of the United States (CLSI, 2023). Minimum inhibitory concentration (MIC) values were categorized as “susceptible (S),” “resistant (R),” and “intermediate (I)”.

### 2.6 Phage isolation

Phages were isolated and purified using an enrichment protocol (Weber et al., 2020) with *P. aeruginosa* PAO1 as the host. Water samples, including hospital wastewater and domestic sewage, were filtered through a 0.22 μm membrane. The filtrate was then mixed with 5 mL of 2 × LB broth and 100 μL of log-phase host culture, followed by incubation at 37 °C with shaking overnight. After incubation, the mixture was centrifuged at 10,000 × g for 5 min, and the supernatant was filtered through a 0.22 μm syringe filter. Phage lysates were purified by five rounds of single-plaque isolation. Titers were determined using the double-layer agar method and stored at 4 °C.

### 2.7 Phage DNA extraction, genome sequencing, and assembly

Phage particles were precipitated with 10% polyethylene glycol 8000 (PEG_8000_) at 4 °C overnight and pelleted by centrifugation (12,000 × g, 30 min). The pellet was resuspended in PBS and treated with DNase I (6 U/mL) and RNase I (50 μg/mL) (Tiangen, Beijing) to remove residual bacterial nucleic acids. ZnCl_2_ (2 M, 20 μL) was added and incubated at 37 °C for 5 min. After centrifugation, pellets were resuspended in TES buffer and digested with 20 μL Proteinase K (Tiangen, Beijing) at 37 °C for 90 min. Then, the DNA was purified by acetic acid potassium treatment, phenol-chloroform extraction and isopropanol precipitation method. The DNA precipitate was washed twice with 70% ethanol, air-dried and dissolved in sterile water. Finally, it was stored at -20 °C.

The phage genome was sequenced through next-generation sequencing (NGS) using an Illumina HiSeq 2000 platform (BGI-Shenzhen, China). Raw reads were quality-filtered with FastQC v0.12.1 (Thambiraja et al., 2026) and Trimmomatic v0.39 (Bolger et al., 2014), followed by genome assembly using SPAdes v3.15.5 (Prjibelski et al., 2020) under default settings. Assembly quality was evaluated with Quast v5.0.2 (Mikheenko et al., 2018), and genome annotation was performed using Pharokka v1.7.0 (Bouras et al., 2023).

### 2.8 Electron microscopy based morphological analysis

Phage morphology was examined by transmission electron microscopy (Yang and Chen, 2024). Briefly, 5 μL of high-concentration purified phage preparation were applied to carbon-supported Formvar grids, negatively stained with 2% phosphotungstic acid, and observed using an H-7000FA transmission electron microscope at 100 kV. Particle dimensions were measured in ImageJ.

### 2.9 Host range determination

The host range of the isolated phages (n = 94) was determined against a panel of 152 *P. aeruginosa* strains. The lytic activity of each phage was assessed using a spot assay. Briefly, 3 μL of phage lysate (1×10^9^ PFU/mL) was spotted onto a lawn of the respective bacterial host and co-cultured on an LB agar plate for 8 h. The interaction between each phage and bacterial strain was recorded as one of the following: “Clear plaques” (formation of distinct and clear plaques), “Turbid plaques” (formation of semi-transparent or frosted-glass-like plaques), or “No plaques” (no visible plaques). A heatmap visualizing the phage-bacteria interaction matrix was generated using the Chiplot online tool.

### 2.10 Efficiency of plating assay

The efficiency of plating was assessed by spot assay. An overnight indicator culture (200 µL, OD_600_ = 0.8) was mixed with 15 mL of soft agar (0.5%, ~ 50 °C) and poured onto LB agar plates to form bacterial lawns. Phage lysates were serially diluted (10^−1^ to 10^−8^), and 3 µL of each dilution was spotted onto the lawns. Plates were incubated at 37 °C for 4–7 h until plaques were visible. Plaques were counted and imaged. All assays were performed in triplicate with three independent biological replicates.

### 2.11 Phage killing curve assay

*P. aeruginosa* strains PAO1 and WK159 were grown overnight in LB broth at 37 °C with shaking (220 rpm). The overnight cultures were cultured at a 1:100 ratio into fresh LB medium and grown to mid-exponential phase (OD_600_ = 0.6). Phage stocks were diluted in PBS to a multiplicity of infection (MOI) of 0.1, based on titers determined by EOP assay. Experiments were performed in 96-well plates (200 µL per well) with an initial OD_600_ of 0.2. The experimental groups included seven individual phage treatment groups, a phage cocktail treatment group, and a negative control group (supplemented with an equal volume of PBS buffer without phage). To compare the complete cocktail with a WK159-targeted sub-cocktail, an additional group containing pap8, pap121, pap131, pap162 was included in the WK159 assay. The complete seven-phage cocktail and the four-phage sub-cocktail were each tested at a total MOI of 0.1. Plates were incubated at 37 °C with continuous shaking in a microplate reader. OD_600_ was recorded every 30 min for 18 h, with a brief shaking step before each measurement. Growth kinetics analysis was plotted using GraphPad Prism v10.4.1, with time on the x-axis and mean OD_600_ values on the y-axis.

### 2.12 Biofilm assays

*P. aeruginosa* PAO1 and six clinical isolates (WK10, WK63, WK121, WK159, WK221, and WK257) were used. Artificial sputum medium (ASM) was used to mimic the respiratory environment. Seven phages (pap1, pap8, pap17, pap104, pap121, pap131, and pap162) were tested individually or as a cocktail prepared at equal proportions (Vashisth et al., 2023).

Biofilm inhibition assay: Groups included individual phages, phage cocktail at different titers, a CIP control (5 μg/mL), and a bacteria-only control. Log-phase cultures (OD_600_ = 1.0) were diluted 1:100 and mixed with the corresponding treatments. Mixtures were added to plates and incubated statically at 37 °C for 24 h. Planktonic cells were removed after incubation. The samples were subjected to methanol fixation, staining with 0.1% crystal violet, and elution with 33% glacial acetic acid, and the OD_570_ value was measured using the microtiter plate assay system (Duan et al., 2021). OD_570_ measured by crystal violet staining represents total biofilm biomass.

Biofilm eradication assay: The strain, phage and medium conditions were identical to those in the biofilm inhibition assay. The 1:100 diluted log-phase bacterial suspension (OD_600_ = 1.0) was added to the plates and incubated statically at 37 °C for 24 h to establish mature biofilms. Planktonic bacteria were removed, and the wells were gently washed with PBS. The corresponding treatment solutions were added, followed by another 24 h of static incubation at 37 °C. The subsequent procedures of fixation, staining, elution and OD_570_ detection were the same as those in the inhibition assay.

Results for PAO1 were presented as the percentage of biofilm inhibition and eradication, both calculated using the formula: Inhibition/Eradication rate (%) = [1 − OD_570_ (treated group)/OD_570_ (bacteria-only control)] × 100. Data are presented as the mean ± SD (n = 4 independent biological replicates). * *p* < 0.05, ** *p* < 0.01, *** *p* < 0.001 comparing each cocktail group with the NC group; ns, not significant. Data analysis was performed using GraphPad Prism v10.4.1.

### 2.13 Phage-antibiotic synergy assay for bacterial

Strains were cultured in LB medium at 37 °C with shaking at 220 rpm until OD_600_ = 0.8, then diluted to approximately 1×10^6^ CFU/mL for later use. The titer of the phage cocktail was determined using EOP and adjusted to gradient concentrations ranging from 10^4^ to 10^9^ PFU/mL. Simultaneously, two-fold serial dilutions of five antibiotics were prepared: polymyxin B (PMB), meropenem (MEM), amikacin (AMK), ceftazidime (CAZ), and ciprofloxacin (CIP). Concentration ranges were 0.125-32 μg/mL for PMB, MEM, AMK, and CAZ, while that for CIP was 1-256 μg/mL. In a 96-well plate, 160 μL of bacterial suspension was mixed with 20 μL of phage cocktail and 20 μL of antibiotic solution (final volume 200 μL per well). Bacterial suspension alone was used as the positive control, and medium alone as the negative control. The plate was incubated at 37 °C for 24 h, and bacterial growth was assessed by monitoring the OD_600_ values of the bacterial culture using an optically based microtiter plate assay system (Synergy H2, Agilent BioTek, Massachusetts, US). The percentage reduction in bacteria was calculated using the formula: Reduction rate (%) = (1 − OD_600_ [Experimental group]/OD_600_ [Control group]) × 100. The resulting data were used to generate a heatmap with GraphPad Prism v10.4.1.

### 2.14 Standardized preparation and quality control protocol for bacteriophage formulations

Bacteriophage preparation amplification: Bacteriophage amplification was performed following a standardized screening and propagation workflow. Briefly, logarithmic-phase host bacteria were inoculated into EZ medium and cultured at 37 °C until the OD_600_ reached approximately 0.2-0.3. Phage suspension was then added and incubation continued until complete bacterial lysis occurred. The resulting primary lysate was subsequently used for large-scale amplification using logarithmic-phase host cultures.

Formulation and purification: Phage formulations were prepared, purified and filling were completed in a GMP-standard facility. 5 mL of 10% Triton X-100 solution (final concentration 0.5%) was added to 100 mL of lysate, followed by incubation at 37 °C with shaking at 150 rpm for 1 h to remove endotoxins. Purification was performed using an ultrafiltration membrane cassette with a molecular weight cutoff of 100 kDa (Vivaflow^®^200, Germany). The membrane cassette was first pre-treated with 250 mL of normal saline, after which the sample was diluted to 200 mL for ultrafiltration. Following a diafiltration ultrafiltration process involving 6 exchanges with normal saline, the solution was finally concentrated to 20 mL. The purified preparation was subsequently filtered through a 0.22 μm sterile membrane and stored at 4 °C.

Quality control: Quality control of the phage formulation included evaluation of appearance, pH, bacteriophage titer, endotoxin level, sterility, and residual Triton X-100. The appearance of the formulation must meet the criteria of a colorless, transparent, or light-yellow clear liquid without visible impurities. pH was measured with a calibrated precision pH meter, with an acceptable range of 6.5-7.5. Phage titers were determined using the double-layer agar method and were required to reach ≥ 10^9^ PFU/mL. Endotoxin levels were measured using limulus amebocyte lysate (LAL) test kits (Bioendo^®^, EC64405). According to the 2024 edition of the Quality Requirements of Phage Preparations Used in Clinical Therapy (Standard No.: T/SHPPA 028-2024), the endotoxin limit is set at < 12.5 EU per 10^7^ PFU. In addition, as recommended in the current Chinese expert consensus on phage therapy (Alliance, Biotechnology, and Microbiology, 2023), the endotoxin limit for non-intravenous formulations is < 250 EU/10^9^ PFU. Sterility testing was performed on Columbia blood agar plates. Triton X-100 residue was quantified by the phenol colorimetric spectrophotometric method: after reacting the sample with a 5% phenol solution, the absorbance was measured at 340 nm, and the concentration was determined using a standard curve. According to the same standard above (T/SHPPA 028-2024), the residual limit should not be exceeding 15.0 μg/mL.

Preparation of bacteriophage cocktail: A defined seven-phage cocktail was formulated for experimental use. Seven individual phage preparations, after passing quality control, were mixed at equal volumetric ratios (1:1:1:1:1:1:1, v/v). Prior to each experiment, the concentrated cocktail was diluted with sterile normal saline to the target concentration.

### 2.15 Animals

Six-week-old male and female BALB/c mice were purchased from Sipeifu Biotechnology Co., Ltd. (Beijing, China). The mice were housed under a 12 h light/dark cycle with ad libitum access to standard chow and sterile water. All animal experiments were performed in an Animal Biosafety Level 2 (ABSL-2) facility. At the end of the experiments, mice were anesthetized by inhalation of 3% isoflurane delivered using a mobile small animal anesthesia system (R520, RWD Life Science, Shenzhen, China) until deep anesthesia was achieved, followed by euthanasia by cervical dislocation. All animal experiments were performed in compliance with the relevant laws and institutional guidelines for the care and use of laboratory animals in China, and in accordance with the ARRIVE guidelines. All procedures involving animals were approved by the Institutional Animal Care and Use Committee (IACUC) of The Third People’s Hospital of Shenzhen [IACUC Approval No. 2024-078-01].

### 2.16 Design of a nose-only inhalation aerosol exposure system for mice

The inhalation chamber system included five key components (Kaur et al., 2020): (a) a medical nebulizer (Yuwell^®^ 407C, China) as the aerosol generator; (b) a rectangular central chamber serving as a four-way flow splitter; (c) flexible silicone tubing connecting the nebulizer to the splitter; (d) sterile single-use inhalation adaptors; and (e) 50 mL sterile centrifuge tubes (Biosharp^®^, China) used as mice restraint chambers. The conical ends of the centrifuge tubes were removed, and a circular opening (≈ 0.9 cm in diameter) was created to fit the mouse snout. The edges were smoothed to prevent injury. Mice were restrained with cotton to allow nasal exposure. A flexible tube (25 cm length, 1.2 cm diameter) connected the nebulizer to the central chamber for aerosol delivery to individual ports. All components were sterilized by autoclaving before use.

Phage titers of the seven-phage cocktail formulation used in the mouse exposure experiments were measured before and after nebulization. A sample was collected immediately before nebulization as the pre-nebulization sample. Based on a previously described aerosol collection method (Astudillo et al., 2018), the generated aerosol was directed into a sterile microcentrifuge tube maintained in an external ice bath, and the resulting condensate was collected as the post-nebulization sample. Titers of the paired pre- and post-nebulization samples were determined using the EOP assay, with *P. aeruginosa* PAO1 as the host strain. Three independent experiments were performed. Throughout the animal studies, the reported phage concentrations and volumes refer to the formulations loaded into the nebulizer rather than the doses inhaled by or deposited in the respiratory tract of individual animals.

### 2.17 Histopathological examination and analysis

Lung, liver, and kidney tissue samples were rinsed in pre-cooled PBS after dissection and fixed in 4% paraformaldehyde at 4 °C overnight. Samples were dehydrated through graded ethanol, cleared in xylene, and embedded in paraffin. Sections (5 μm) were prepared and stained with H&E for morphological analysis (Zhou et al., 2024).

Histopathological assessment was conducted by Wuhan Servicebio Technology Co., Ltd (Wuhan, China). Images were captured at 20 × and 200 × magnification. Pathological features, including congestion, hemorrhage, edema, degeneration, necrosis, hyperplasia, fibrosis, and inflammatory infiltration, were recorded. Pathological injuries in lung (Matute-Bello et al., 2011), liver (Goodman, 2007), and kidney (Mahajan and Gowrishankar, 2024) tissues were assessed using established semi-quantitative scoring systems. Scores were based on lesion severity and distribution, and a total score was calculated.

### 2.18 Hematology and serum biochemistry

Blood samples were collected from the orbital venous plexus of all mice under anesthesia with 3% isoflurane. The collected blood was placed in anticoagulant tubes containing dipotassium ethylenediaminetetraacetic acid (EDTA) and analyzed for hematological parameters using a hematology analyzer (BC-2800Vet, Mindray, Shenzhen, China). Blood was collected and transferred into a sterile tube or a plasma separation tube containing lithium heparin anticoagulant (light green cap). After centrifugation at 2000 × g for 15 min, the supernatant plasma was separated and subjected to biochemical analysis using a fully automated clinical chemistry analyzer (Chemray 240, Rayto, Shenzhen, China).

### 2.19 Evaluation of bacteriophage therapeutic efficacy

Six-week-old BALB/c mice (18–21 g, half male and half female) were acclimatized for one week in an ABSL-2 facility. The pneumonia model was established by intranasal inoculation with *P. aeruginosa* WK159 (1 × 10^8^ CFU/mL, 50 μL per mouse) (Yang et al., 2021). 4 h post-infection, mice were randomly divided into four groups (n = 12, 6 males and 6 females per group): an infection control group receiving normal saline (Infection control), and three treatment groups receiving a nebulized phage cocktail at low- (Cocktail LD, 5 × 10^7^ PFU/mL), medium- (Cocktail MD, 5 × 10^8^ PFU/mL), or high-dose (Cocktail HD, 5 × 10^9^ PFU/mL). Treatments (0.5 mL per mouse) were administered once daily for 7 days via oro-nasal nebulization (Bolsan, 2024). Survival and body weight were monitored daily. For histopathology, tissues were fixed in 4% paraformaldehyde, embedded in paraffin, sectioned, and stained with hematoxylin and eosin (H&E). To evaluate bacterial clearance, an additional cohort of mice (n = 12, 6 males and 6 females per group) received either normal saline (Infection control) or the high-dose phage cocktail (Cocktail HD). At 24 h after treatment, heart, liver, spleen, lung and kidney tissues were collected aseptically, homogenized, serially diluted, and plated on LB agar.

### 2.20 Evaluation of bacteriophage safety profile following long-term administration

Six-week-old BALB/c healthy (non-infected) mice (18–21 g, half male and half female) were housed under ABSL-2 conditions for one week. Mice were then randomly assigned to two groups (n = 12 per group): a saline control (NaCl Control) and a high-dose phage cocktail group (Cocktail HD, 5 × 10^9^ PFU/mL). Animals received daily oro-nasal nebulization (0.5 mL per mouse) for 14 days (Day 0-14), followed by a 14-day observation period (Day 14-28) (Chan et al., 2025). Blood samples were collected before treatment (Day 0), during the dosing period (Day 3, 7, 14), and at the end of the observation phase (Day 28). Hematological and biochemical analyses were conducted following the procedures detailed in the previous section. At the study endpoint (Day 28) after long-term administration, mice were euthanized, and tissues including lung, liver, and kidney were collected for histopathological processing and analysis according to the methods described above.

### 2.21 Biodistribution study of phage cocktail following single-dose administration

A cohort of BALB/c mice (n = 12, 6 males and 6 females per group) received a single oro-nasal nebulization of either saline (NaCl control), a low-dose phage cocktail (Cocktail LD, 5 × 10^7^ PFU/mL), or a high-dose phage cocktail (Cocktail HD, 5 × 10^9^ PFU/mL). Mice were euthanized at 2, 4, 8, and 24 h post-administration, and heart, liver, spleen, lung, and kidney tissues were aseptically collected and weighed. Each tissue sample was homogenized in 1 mL of PBS buffer using grinding beads in a high-throughput tissue homogenizer. The homogenates were centrifuged at 4 °C (8000 × g, 10 min), and the supernatants were serially diluted in PBS buffer. Phage titers in each organ were quantified using the double-agar overlay method with *P. aeruginosa* PAO1 as the host strain.

### 2.22 Statistical analysis

Statistical analyses were performed using GraphPad Prism v10.4.1. Data are presented as mean ± standard deviation (SD). Survival data were analyzed using the Kaplan-Meier method with the log-rank test. Longitudinal data were analyzed by two-way ANOVA with Tukey’s multiple-comparison test. For comparisons between two groups, the Mann-Whitney U test was used. For comparisons among multiple groups, the Kruskal-Walli’s test followed by Dunn’s multiple-comparison test was used. A two-tailed *p* < 0.05 was considered statistically significant. In the figures, *p < 0.05, **p < 0.01, ***p < 0.001 and ****p < 0.0001; ns indicates no statistical significance.

## 3 Results

### 3.1 Genomic characterization and antibiotic resistance profile of clinical *P. aeruginosa* isolates

We collected a total of 152 clinical isolates of *P. aeruginosa* (**Figure 1**). Most isolates were obtained from sputum specimens (92/152, 59.9%), whereas the remaining isolates were obtained from bronchoalveolar lavage fluid (BALF), blood, wound exudates, cerebrospinal fluid, urine, and other sites. Nearly half of the isolates were from patients with pneumonia or lung cancer (76/152, 50.0%), while the rest associated with burn injuries, septicemia, leukemia, diabetes, and other conditions.

**Figure 1:**
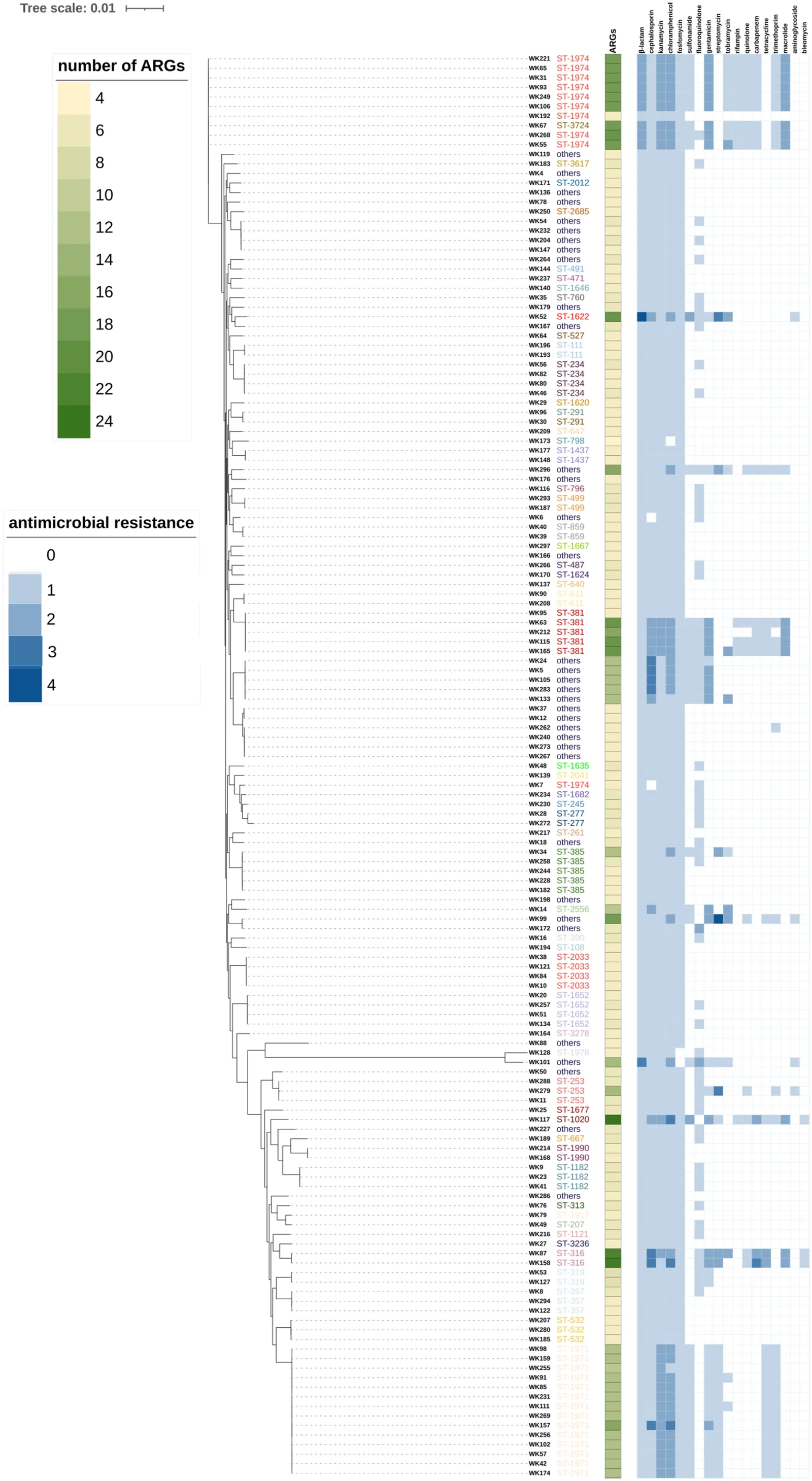
Phylogenetic tree of strains with a heatmap showing antimicrobial resistance gene (ARGs) profiles. The left panel shows the whole-genome phylogenetic tree of the analyzed strains (scale bar = 0.01), annotated with strain IDs and MLST profiles. The number of ARGs in each strain is indicated by a color gradient (light yellow to dark green; range: 4–24). The heatmap displays the distribution and abundance of ARGs associated with different antibiotic resistance phenotypes across strains (range: 0–4 per type).

All 152 *P. aeruginosa* strains were sequenced by whole-genome, and then the core genome phylogenetic tree was constructed to clarify their evolutionary relationship. The resulting phylogenetic tree was divided into three major clusters, reflecting the genomic diversity of *P. aeruginosa* within our cohort. Antibiotic resistance genes (ARGs) were identified across all genomes and visualized as a heatmap and were further classified by antibiotic class and mechanism of action. CRPA represented 26.3% (40/152) of the isolate collection, with ST1971 (15/152, 9.7%) and ST1974 (10/152, 6.6%) emerged as the most dominant STs. The isolates analyzed here demonstrate broad clinical and genomic diversity across multiple anatomical sources in hospitalized patients. Targeting these strains with lytic bacteriophages may contribute to the development of effective treatment strategies for MDR *P. aeruginosa* respiratory infections.

### 3.2 Screening and characterization of phage cocktail components

A library of 94 phage isolates was tested against 152 clinical *P. aeruginosa* strains representing diverse serotypes (O1-O7, O9-O13, and non-typable isolates) (**Figure 2A**). A spot assay was then performed to generate an interaction matrix comprising 14,288 phage-bacterium combinations for evaluation of host coverage and lytic activity. Based on these results, a phage cocktail was designed to achieve broad host coverage with strong lytic activity. Four phages with wide host ranges and clear plaques (pap162, pap17, pap8, pap131) were selected as the core components. Three additional phages with complementary host spectra (pap104, pap121, pap1) were included to improve coverage, particularly for serotypes O5 and O11. The final cocktail consisted of one lipopolysaccharide (LPS)-targeting phage and six type IV pilus (T4P)-targeting phages (**Supplementary Figure 1**). Then, we used the spot assay to evaluate the lytic activity of each individual phage and the full cocktail against all 152 bacterial strains (**Figure 2B**). The bacteriophage cocktail can lyse 89.61% of the tested strains, with clear plaques observed in 74.03% of cases (**Figure 2C**). Overall, the cocktail displayed broader host coverage and stronger lytic activity than any individual phage, with no evidence of interference among the selected phages.

**Figure 2:**
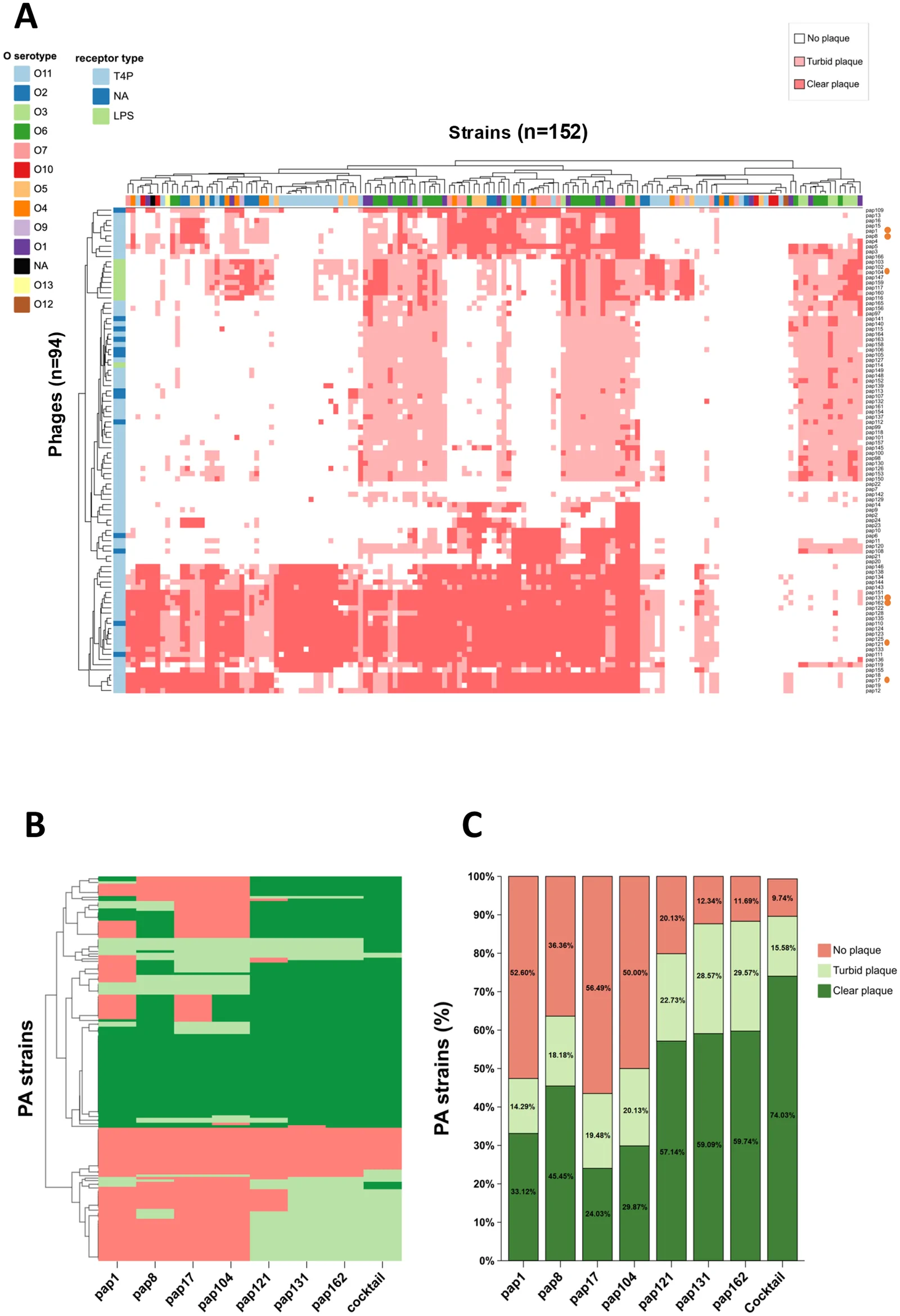
Characterization of phage lytic spectra against *P. aeruginosa* strains. **(A)** Heatmap showing the lytic activity of 94 phages against 152 *P. aeruginosa* strains. Receptor types (determined on PAO1) are labeled on the left: T4P: type IV pilus; LPS: lipopolysaccharide; NA: not applicable. O-antigen types of the strains are indicated at the top. **(B)** Heatmap showing the lytic patterns of individual phages (pap1, pap8, pap17, pap104, pap121, pap131, pap162) and the phage cocktail against *P. aeruginosa* strains. **(C)** Stacked bar chart quantifying the lytic coverage of individual phages and the phage cocktail against *P. aeruginosa* strains. The proportions of strains exhibiting no plaque, turbid plaque, and clear plaque are represented in red, light green, and dark green, respectively.

The selected phages were further characterized by morphological, genomic, and biological analyses. Plaque morphology varied in size and appearance across phages (**Supplementary Figure 2A**). Transmission electron microscopy (**Supplementary Figure 2B**) confirmed that all phages exhibited typical icosahedral heads and tailed structures, classifying them within members of the class *Caudoviricetes*. Whole-genome sequencing showed that double-stranded DNA genomes with conserved core functional modules in all seven phages, with no antibiotic resistance genes, lysogeny-associated genes, or virulence factors detected (**Supplementary Figure 2C**). The key biological characteristics of these bacteriophages were further evaluated, including the stability under different pH, temperature and MOI conditions. All bacteriophages maintain a high titer (∼10^8^-10^10^ PFU/mL) in the pH range of 4-11 (**Supplementary Figure 3A**). Titers were stable between 4 °C and 50 °C, declined in some phages (e.g., pap8) at 60 °C, and decreased sharply at 70-80 °C (**Supplementary Figure 3B**). In the range of 0.0001 to 10 MOI, the bacteriophage titers always maintained a high level (10^10^-10¹¹ PFU/mL), while the situation of pap17 changed only slightly and peaked at an MOI of 10 (**Supplementary Figure 3C**). These results show that the seven phages have good biological characteristics, supporting their potential for therapeutic application against MDR *P. aeruginosa*.

### 3.3 Phage-mediated inhibition and eradication of *P. aeruginosa* biofilms

To evaluate the anti-biofilm effect potential of the bacteriophage library in this study, seven individual phages (pap1, pap8, pap17, pap104, pap121, pap131, and pap162) and their phage cocktail were examined the biofilm inhibition and eradication effects of against the *P. aeruginosa* reference strain PAO1, with CIP as a positive control (**Figure 3A**). In the biofilm inhibition assays, pap104 showed the strongest inhibitory effect (~ 77%), followed by pap121 (~ 75%) and pap17 (~ 61%). The remaining phages exhibited moderate inhibition. It is worth noting that the phage cocktail displayed stronger inhibitory activity than all single phages tested (~ 90%). In biofilm eradication assays, the phage cocktail showed the highest activity (~ 63%), exceeding that of all individual phages. Pap104 showed relatively strong eradication (~ 55%), whereas pap162 and pap131 exhibited moderate effects (~ 34%). In contrast, CIP inhibited biofilm formation but showed minimal activity against established biofilms (< 7% eradication).

**Figure 3:**
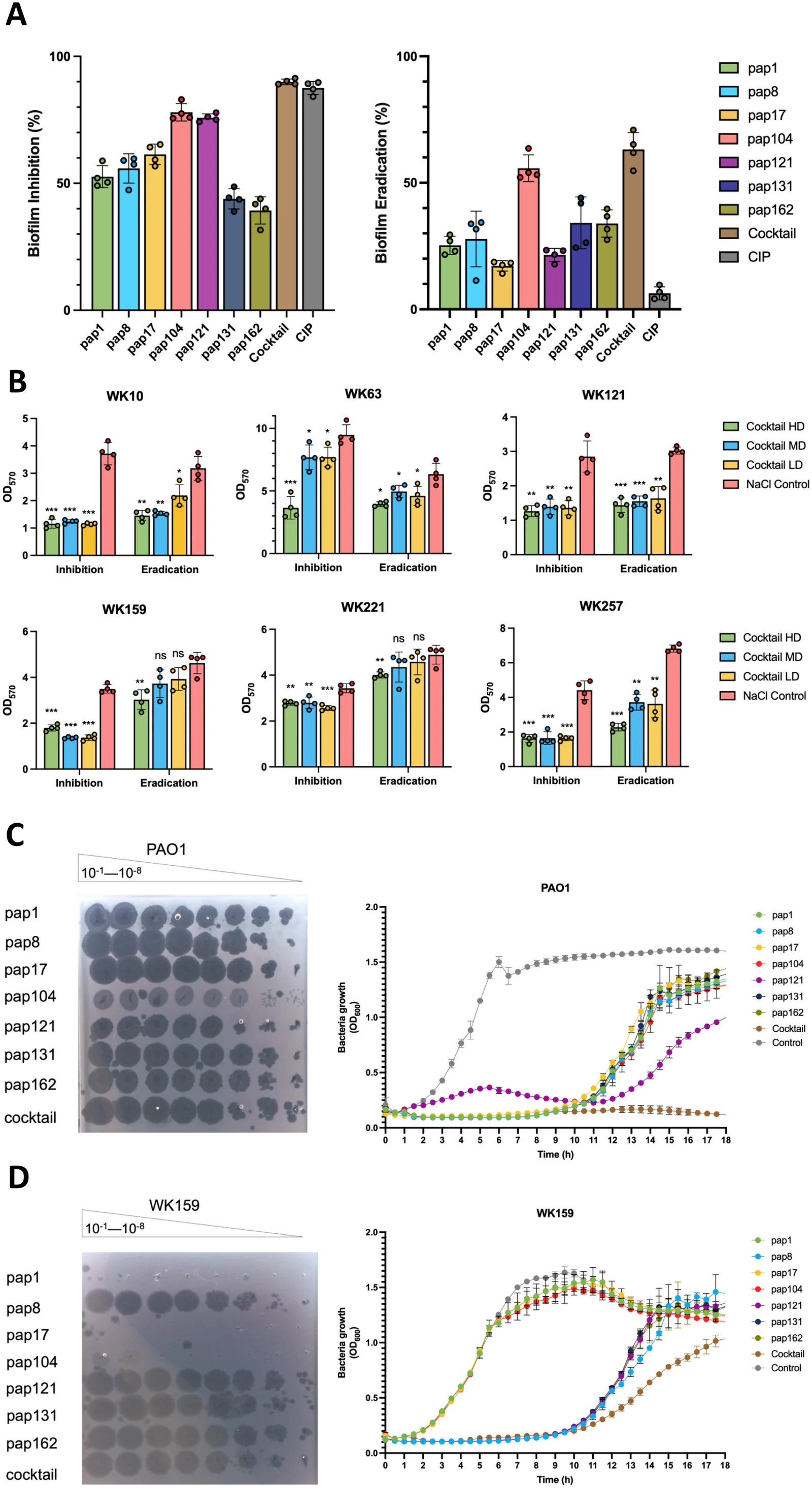
*In vitro* efficacy of single phages and phage cocktails against *P. aeruginosa* biofilms and cultures. **(A)** Biofilm inhibition and eradication by single phages and cocktails. Bar charts showing the percentage of biofilm inhibition (left) and eradication (right) by individual phages (pap1, pap8, pap17, pap104, pap121, pap131, pap162), phage cocktail, and CIP against *P. aeruginosa* PAO1 biofilms. **(B)** Biofilm inhibition and eradication in clinical isolates. Bar charts of OD_570_ values, representing biofilm biomass, for six clinical *P. aeruginosa* isolates treated with the phage cocktail at high-dose (HD), medium-dose (MD), low-dose (LD), or a NaCl control. Data are presented as mean ± SD (n =4). **(C)** Activity against *P. aeruginosa* PAO1. Left: Spot assay showing the lytic activity of single phages and the cocktail against *P. aeruginosa* PAO1 at serial dilutions. Right: Growth kinetics analysis (OD_600_, 18h) of PAO1 cultures treated with single phages, the cocktail, or a control, demonstrating the inhibitory effect of phages on planktonic growth. Data are presented as mean ± SD (n =4) **(D)** Activity against *P. aeruginosa* WK159. Left: Spot assay showing lytic activity of single phages and the cocktail against the clinical isolate WK159. Right: Growth kinetics analysis (OD_600_, 18h) of WK159 cultures treated with single phages, the cocktail, or a control, illustrating the impact of phages on planktonic growth of this clinical strain.

We next assessed the anti-biofilm activity of the phage cocktail against six clinical *P. aeruginosa* isolates (WK10, WK63, WK121, WK159, WK221, and WK257) derived from sputum, bronchoalveolar lavage fluid, or deep venous catheters of patients with pneumonia (**Supplementary Table 1**). Biofilm assays were performed in artificial sputum medium to mimic the respiratory microenvironment *in vitro*. The phage cocktail was tested at high (Cocktail HD), medium (Cocktail MD), and low (Cocktail LD) titers, with NaCl treatment as the control (**Figure 3B**). Across all isolates, phage-treated groups showed lower OD_570_ values than the NaCl control, with the high-titer cocktail (HD) consistently giving the lowest OD_570_ values. The phage cocktail could also disrupt established biofilms of several clinical isolates. Biofilm biomass decreased at all doses in WK121 and WK257, while HD and MD were more effective than LD in WK63. The phage cocktail inhibited biofilm formation and disrupted established biofilms in both reference and clinical *P. aeruginosa* isolates.

### 3.4 Lytic activity of individual phages and phage cocktail against *P. aeruginosa*

To further assess antibacterial activity, the antibacterial performance of individual phages and the phage cocktail was evaluated using the reference strain PAO1 (**Figure 3C**). Plaque assays showed comparable initial titers across individual phages, and the cocktail exhibited a similar overall titer. In time-kill assays, single phage treatment resulted in only transient inhibition of PAO1 growth, with bacterial regrowth observed after approximately 10 h. In contrast, the cocktail maintained a low bacterial density throughout the 18 h culture period (**Figure 3C**), suggesting a more sustained antibacterial effect.

To evaluate activity against a clinically relevant drug-resistant strain, the MDR isolate WK159 was further analyzed (**Figure 3D**). This strain was isolated from a patient with pneumonia and exhibited resistance to multiple antibiotics, including carbapenems and polymyxins (**Supplementary Table 2**; genomic features shown in **Supplementary Figure 4**). Host range analysis showed that only pap8, pap121, pap131, and pap162 formed clear plaques on WK159, whereas the other phages displayed weak lytic activity. In time-kill assays, the cocktail delayed bacterial growth and maintained a low bacterial density than the individual phage treatments during the incubation period (**Figure 3D**). We next compared the antibacterial activity of the complete seven-phage cocktail and the four-phage sub-cocktail, which contains pap8, pap121, pap131, and pap162, against WK159 at the same total MOI of 0.1. Both cocktails delayed bacterial growth compared with the no-phage control. No clear difference in bacterial growth suppression was observed between the four-phage sub-cocktail and the complete seven-phage cocktail under the tested conditions (**Supplementary Figure 5**).

### 3.5 *In vivo* therapeutic efficacy of the phage cocktail in a murine pneumonia model

The therapeutic efficacy of the phage cocktail was further assessed *in vivo* using a murine model of pneumonia established by intranasal infection with *P. aeruginosa* strain WK159. Infected mice developed typical symptoms of bacterial pneumonia, including weight loss, reduced food intake, hypothermia, decreased activity, ruffled fur, tachypnea, and hunched posture.

Based on this model, we further assessed the *in vivo* protective efficacy of the bacteriophage cocktail (see **Figure 4A** for the experimental timeline). After intranasal administration of WK159 on day 0 (D0), the mice received phage intervention for seven consecutive days, and the endpoint analysis was carried out on D7. Gross examination showed swollen and hyperemic lung lobes with multiple abscesses (**Figure 4B**), indicating successful model establishment. Survival analysis demonstrated that phage treatment significantly improved survival outcomes. The high-dose cocktail group (Cocktail HD) had a higher survival rate than the saline control (Infection control) (**Figure 4C**, *p* < 0.0001). In contrast, the medium- and low-dose groups (Cocktail MD and LD) provided only limited protective effects (**Figure 4C**, *p* < 0.05).

**Figure 4:**
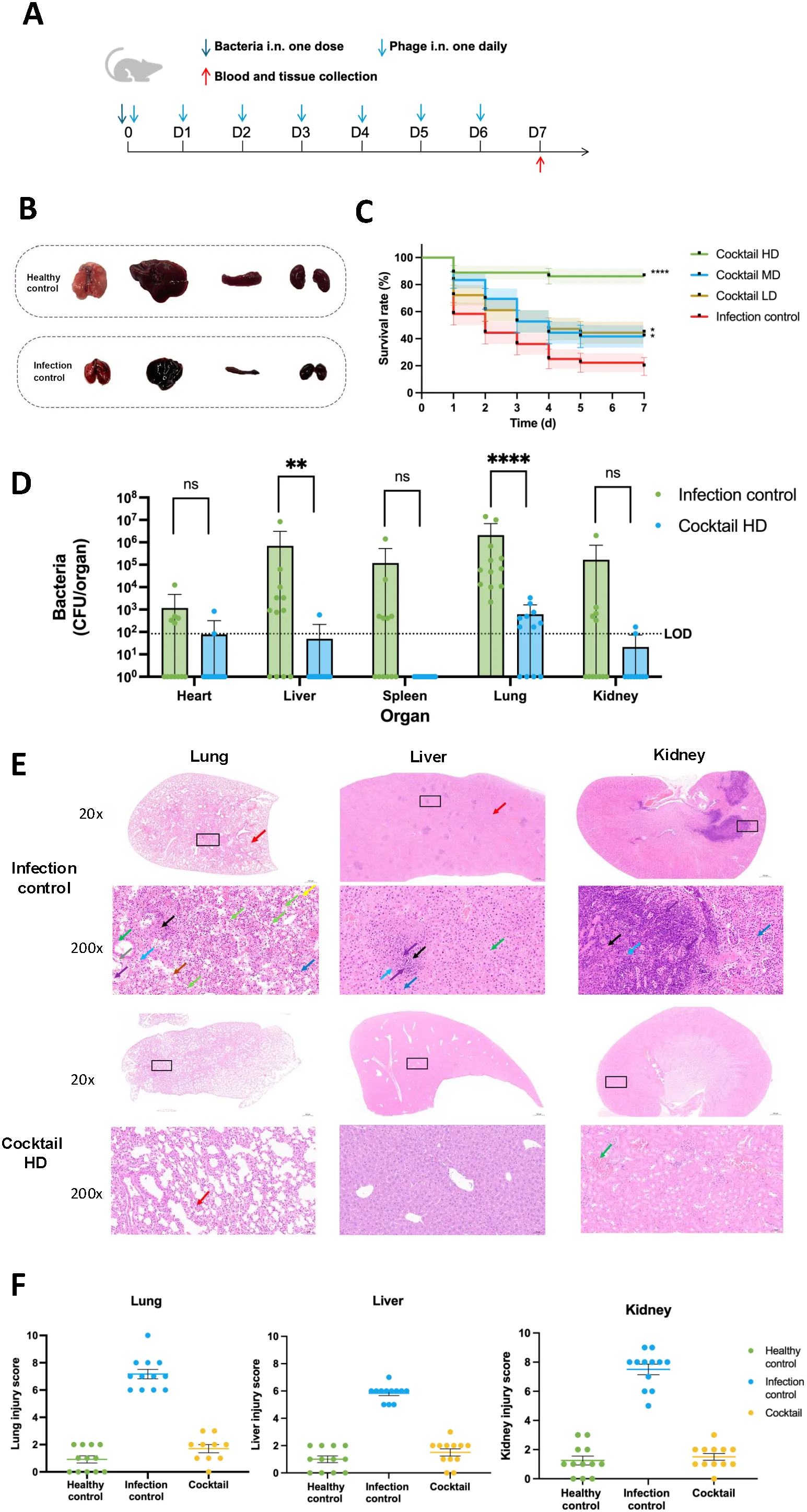
*In vivo* therapeutic efficacy of phage cocktail against MDR *P. aeruginosa* WK159 infection in mice. **(A)** Schematic diagram of the experimental procedure. Mice were challenged with a single dose of MDR *P. aeruginosa* WK159 on day 0 (D0), followed by 7 consecutive days of phage cocktail administration, with day 7 (D7) set as the experimental endpoint. **(B)** Gross morphology of major organs (lung, liver, spleen, kidney) in the Healthy control group and Infection control group. **(C)** Survival curves of mice (n = 12 per group) treated with different doses of the phage cocktail (Cocktail HD, MD, and LD) or Infection control over a 7-day period post-infection. ****p < 0.0001, *p < 0.05 vs. Infection control. **(D)** Bacterial loads (CFU/organ) in the heart, liver, spleen, lung, and kidney of mice in the Infection control group and Cocktail HD group. **(E)** Histopathological sections (20 × and 200 × high magnification) of the lung, liver, and kidney in the Infection control group and Cocktail HD group. Black boxes indicate regions shown at higher magnification. Arrows indicate pathological lesions: ① -Lung: Infection control group—granulocyte infiltration in alveolar walls (blue arrows), necrotic cell debris (black arrows), basophilic granules (light blue arrows), alveolar wall capillary congestion (yellow arrows), exudation of macrophages, lymphocytes, granulocytes, and eosinophilic material in alveolar cavities (light green arrows), alveolar hemorrhage (brown arrows), irregularly arranged bronchiolar epithelial cells (green arrows), perivascular edema (gray arrows), occasional lymphocytic infiltration (purple arrows), mild vascular congestion (red arrows). Cocktail HD group—mild granulocyte infiltration in alveolar walls with focal mild alveolar wall thickening and widened alveolar septa (red arrows). ② -Liver: Infection control group—hepatocellular edema (green arrows), vacuolar degeneration of hepatocytes (blue arrows), focal hepatocellular necrosis (black arrows), lymphocytic infiltration (purple arrows), basophilic granules (light blue arrows), massive vascular congestion (red arrows). Cocktail HD group—mild hepatocellular edema (green arrows). ③ -Kidney: Infection control group—necrotic foci (black arrows), lymphocytic infiltration (purple arrows). Cocktail HD group—mild interstitial vascular congestion (green arrows). **(F)** Tissue injury scores of the lung, liver, and kidney in Healthy control, Infection control and Cocktail groups.

Bacterial loads were measured to assess pathogen clearance. Compared with the control group, mice treated with the high-dose phage cocktail (Cocktail HD) exhibited a significant reduction in bacterial loads in the lungs (*p* < 0.0001) and liver (*p* < 0.01). Bacterial counts in the heart, spleen, and kidneys also showed a modest decreasing trend but did not reach statistical significance (**Figure 4D**). Histopathological analysis showed tissue damage in the control group, including alveolar wall thickening, inflammatory cell infiltration, congestion, and edema in the lung, liver, and kidney (**Figure 4E**). In contrast, the Cocktail HD group showed markedly reduced pathological damage and more complete tissue structure. The quantitative injury score (**Figure 4F**) showed that the lung, liver, and kidney injury scores in the infection model group (Infection control) were significantly higher than those in the normal control group (Healthy control). In contrast, the score of the bacteriophage treatment group (Cocktail) was significantly reduced, close to the level of the Healthy control group. This shows that bacteriophage intervention effectively reduces the histopathological damage caused by infection. Overall, the phage cocktail showed significant therapeutic efficacy in the murine pneumonia model, such as improving survival rate, reducing bacterial load in organs and alleviating histopathological damage.

### 3.6 *In vivo* safety profile of inhaled phage cocktail in mice

To evaluate the *in vivo* safety of inhaled phage therapy, the high-dose phage cocktail (Cocktail HD) was selected for subsequent safety evaluation based on the therapeutic efficacy observed in the preceding experiments. Mice received daily nebulized treatment with the phage cocktail or saline for 14 days, followed by a 14-day observation period (**Figure 5A**). Blood samples were collected on days 0, 3, 7, 14, and 28, and tissues were harvested on day 28 for comprehensive safety analyses.

**Figure 5:**
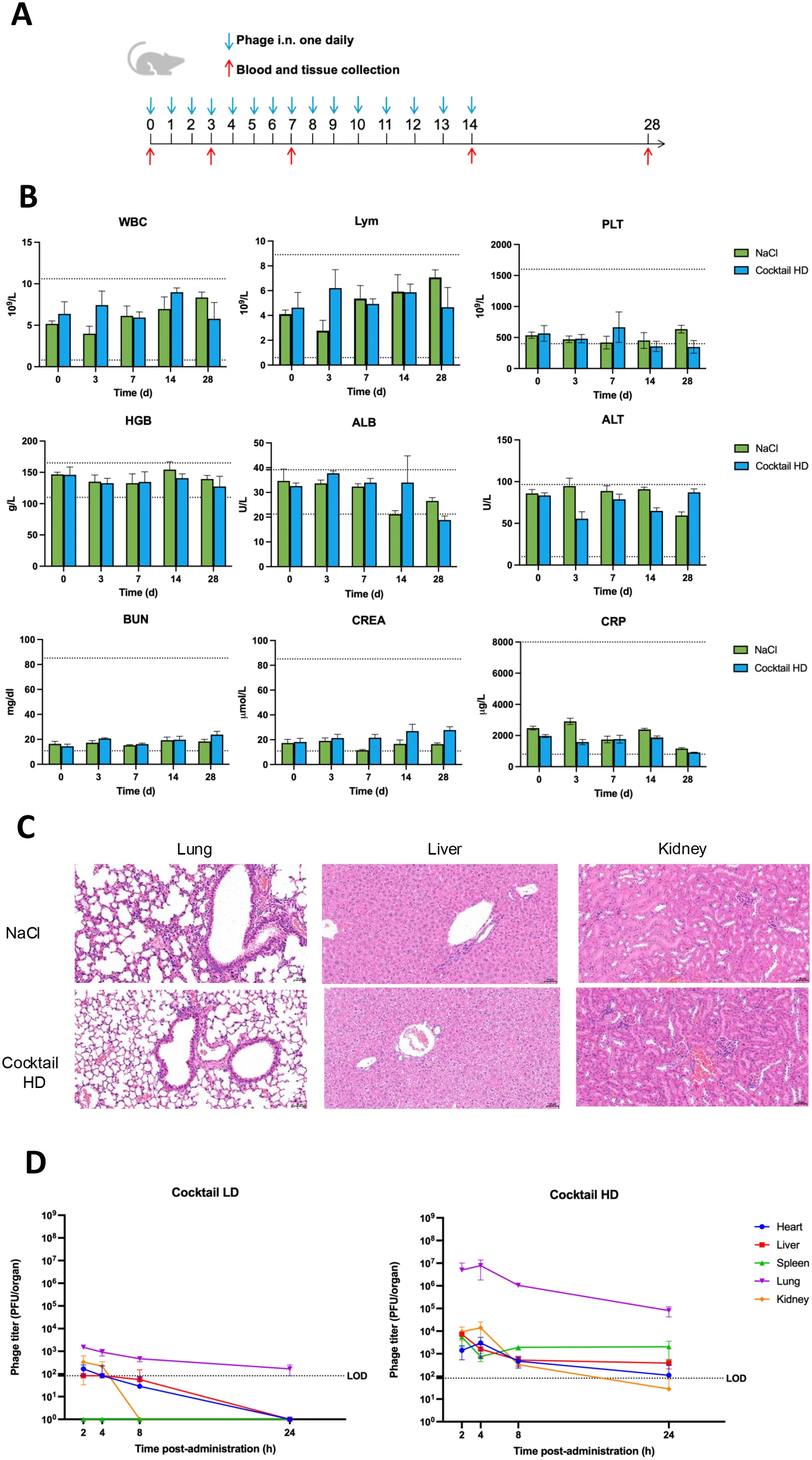
*In vivo* safety and biodistribution of phage cocktail in mice. **(A)** Schematic diagram of the experimental procedure. Healthy (non-infected) mice were randomly divided into a normal saline control group (NaCl) and a high-dose phage cocktail group (Cocktail HD). Day 0 (pre-administration) served as the baseline for blood sample collection. Mice in both groups received intranasal nebulization once daily from Day 0 to Day 14, with the Cocktail HD group receiving phage cocktail and the NaCl group receiving an equal volume of normal saline. A follow-up observation period was conducted from Day 14 to Day 28. Blood samples were collected on Days 3, 7, 14, and 28. On Day 28 (experimental endpoint), both blood and tissue samples were collected. **(B)** Hematological and biochemical parameters measured at different time points (0, 3, 7, 14, 28 d) in mice from the NaCl and Cocktail HD groups. **(C)** Histopathological examination of mouse lung, liver and kidney tissues: HE-stained sections from the two groups [as shown in panel **(A)**] were observed at 200 × magnification. **(D)** Tissue distribution of phages. Phage titers (PFU/organ) in the heart, liver, spleen, lung, and kidney at 2, 4, 8, and 24 h after a single intranasal nebulization of Cocktail LD or Cocktail HD phage cocktail. LOD, limit of detection.

Following long-term treatment, no significant differences were observed between the HD group and the saline control in hematological and biochemical parameters (**Figure 5B**). Peripheral blood indices (WBC, Lym, Neu), and inflammatory markers (PCT, CRP) remained stable throughout the study. Similarly, liver and kidney function markers (ALB, ALT, CREA) also stayed within normal ranges at all time points, indicating no detectable hematological, inflammatory, or hepatorenal toxicity. To further assess the acute response within a shorter time window, we monitored the dynamic changes in hematological and biochemical parameters within 24 h after a single administration (**Supplementary Figure 6**). Compared with the saline control group, both low- and high-dose phage cocktail groups exhibited no significant alterations in peripheral blood indices (WBC, Neu, Lym, HGB), inflammation marker (CRP), or liver- and kidney-related biochemical markers (ALT, BUN, CREA) at any time point during the 24h period.

We next evaluated the biodistribution of the phage cocktail in major organs within 24 h following a single administration (**Figure 5D**). In the low-dose group, phage titers were detectable in the sampled organs at 2 h post-administration and gradually decreased to below the limit of detection (LOD) over time, whereas phages remained undetectable in the spleen at all time points. In the high-dose group, higher initial phage titers were observed across organs, peaking at ~ 4 h. Lung tissue exhibited slightly higher initial phage levels than other organs, but phage titers subsequently declined in all tissues. These findings show transient organ distribution of phages with subsequent clearance and no evidence of accumulation.

## 4 Discussion

MDR *P. aeruginosa* is a major pathogen in respiratory infections because of its high pathogenicity (Reynolds and Kollef, 2021), adaptive mutability (Pei et al., 2025), and strong biofilm formation ability (Moser et al., 2021; Jones and Wozniak, 2017), and is associated with high mortality rates (Shi and Xie, 2023; Li et al., 2024; Xiao et al., 2025). Phage therapy has attracted increasing attention as a potential treatment for bacterial infections, especially those caused by antimicrobial-resistant pathogens. However, its clinical application still requires further optimization and standardization. In this study, we developed a nebulized phage cocktail targeting MDR *P. aeruginosa* through a systematic screening strategy (“broad coverage with strong lysis and receptor complementarity”). The cocktail was evaluated for antibacterial spectrum, anti-biofilm activity, *in vivo* efficacy, and safety. The results suggest that this approach may have potential as a preclinical therapeutic strategy for refractory respiratory infections caused by MDR *P. aeruginosa*.

Previous studies have found that biofilm formation is a key mechanism for *P. aeruginosa* to evade antibiotic treatment and establish persistent infections (De Jonge et al., 2019; Rawat et al., 2025). Phage therapy has attracted attention because of its inherent anti-biofilm potential. This potential is primarily attributed to their ability to not only penetrate the biofilm matrix (Meneses et al., 2025), but also to produce enzymes that degrade biofilm components, and target persistent bacterial cells within these communities. Disruption of the matrix can release embedded bacteria and shift them to a planktonic state, where they become more susceptible to phage attack and clearance (Rahman et al., 2011). To evaluate the anti-biofilm efficacy of bacteriophages under physiologically related conditions, anti-biofilm activity was evaluated using an artificial sputum medium that mimics the respiratory environment. The phage cocktail showed clear activity against both forming and established biofilms and performed better than CIP under the tested *in vitro* conditions, supporting its potential for further evaluation in biofilm-associated *P. aeruginosa* infections. Some phages can target persisted cells within biofilms formed in the airways of patients with ventilator-associated pneumonia. These cells remain in a dormant, non-dividing state and are less susceptible to antimicrobial agents. Once bacterial growth restarts, phages can enter the lytic cycle and eliminate them, which may help limit infection recurrence. Among the phages tested, the LPS-targeting phage pap104 showed relatively strong activity. This may be related to improved penetration into the biofilm or more effective disruption of its structure. Collectively, these findings indicate that phages retain substantial anti-biofilm potential even under conditions that closely resemble the *in vivo* pulmonary environment. However, crystal violet staining cannot distinguish viable from non-viable cells within the biofilm. Future studies should combine viable cell counts with live/dead fluorescence staining to assess bacterial viability. Confocal imaging combined with appropriate fluorescent staining could also be adopted to distinguish bacterial cells from matrix components and further characterize biofilm structure.

Phage therapy currently remains largely empirical, with diverse dosing regimens including single phages or phage cocktails, different administration routes, and differences in dose, frequency, and treatment duration, as well as the use of antibiotics (Mitropoulou et al., 2022). Clinical data regarding optimal phage delivery strategies for pulmonary infections remain limited. In this study, we therefore explored an inhalation-based therapeutic regimen in a murine pneumonia model. The survival benefit and reduced bacterial burden observed after high-dose nebulized treatment support further evaluation of this delivery approach in clinical settings. Nebulization caused only a small reduction in the titer of the seven-phage cocktail (∼ 0.15 log loss) in our study, as shown in **Supplementary Table 3**. However, the aerosol particle-size distribution generated by the complete nebulization system was not characterized, and neither the actual inhaled dose nor the lung-deposited dose was quantified. Therefore, the concentrations and volumes reported in this study represent the phage formulation loaded into the nebulizer rather than the amount inhaled by or deposited in the respiratory tract of each animal. The efficiency of phage delivery to the lungs could not be precisely assessed. Future studies should characterize the aerosol output, particle-size distribution, and regional lung deposition of viable phages under specific devices and formulation conditions used. Detection of phages in the bloodstream was consistent with their presence in circulation-associated organs such as the spleen, as shown in the biodistribution analysis. This suggests that systemic dissemination may contribute to bacterial clearance beyond the lungs and indicates potential efficacy against bacteremia secondary to pulmonary infection. In addition, bacteriophages showed a transient distribution in the body: the low-dose group drops to the lower limit of detection within 24 h, and the high-dose group only retains a small amount in some tissues. However, because aerosol deposition and pulmonary exposure were not quantified, these data do not establish an optimal dosing interval. Further pharmacokinetic and dose-deposition studies are required to determine the amount of viable phage reaching the lungs and to optimize the dosing frequency.

The complete seven-phage cocktail was used in the animal experiments because it was developed as a fixed broad-host-range formulation based on activity across the clinical isolate panel, rather than specifically for WK159. WK159 was selected as the challenge strain because the cocktail showed robust *in vitro* antibacterial activity against this MDR clinical isolate. However, only a subset of the cocktail components formed clear plaques on WK159. Therefore, this single-strain model did not fully evaluate the contribution of all seven phages. Pap104, which showed no lytic activity against WK159, was the only LPS-targeting phage in the cocktail. In contrast, the four phages that formed clear plaques on WK159 (pap8, pap121, pap131, and pap162) all target T4P. Thus, this model cannot determine whether targeting different receptors could limit bacterial resistance development. Presumably, repeated treatment therefore mainly imposed T4P-directed selective pressure may have favored the emergence of T4P-deficient cross-resistant mutants. Notably, bacterial colonies remaining in the lungs were not collected at Day 7, so their phage susceptibility and T4P-related phenotypes were not examined *in vivo*. However, we did an *in vitro* time-kill experiment, of which the results indicated that resistant bacteria would emerge after 14h (**Supplementary Figure 5**). Future studies should evaluate the complete cocktail against multiple MDR *P. aeruginosa* isolates with different phage susceptibility profiles. Residual bacteria should also be collected after repeated treatment to assess their phage susceptibility and receptor-related phenotypic changes. In the additional time-kill assay, bacterial growth resumed at approximately the same time in the seven-phage and four-phage groups (**Supplementary Figure 5**). The addition of the three weakly active phages therefore neither delayed nor accelerated bacterial regrowth under the tested conditions. Thus, no detectable synergistic benefit or overall inhibitory effect was observed. However, the assay did not directly assess phage adsorption or receptor binding. Therefore, whether receptor competition occurred could not be directly determined. Moreover, the potential immunological burden associated with repeated administration of the complete formulation should be considered. Phage-specific immune responses, including neutralizing antibodies, may influence phage persistence *in vivo* and therapeutic activity during repeated treatment. Future studies should compare pulmonary and systemic immune responses, neutralizing antibody production, and the *in vivo* persistence of different phage formulations during repeated inhalation both in animal model and in clinical settings.

In recent years, many case reports on phage therapy have been published (Mitropoulou et al., 2022; Aslam et al., 2019; Teney et al., 2024), and multiple clinical trials are in progress. Among these, the duration of phage therapy varies greatly, ranging from 2 days to over 12 months (Mitropoulou et al., 2022). According to clinical trial experience, a 14-day administration period is commonly regarded as an effective long-term therapeutic plan in patient populations (Tan et al., 2021; Chen et al., 2022). Based on this, we assessed the safety of prolonged pulmonary administration of the phage cocktail. Long-term safety assessment after 14 days of treatment followed by a 14-day observation period showed no abnormalities in hematological parameters, inflammatory markers, or liver and kidney function, and no histopathological damage in major organs. Similarly, no significant changes were observed in hematological or biochemical parameters within 24 h after a single exposure, suggesting good tolerability under the proposed treatment regimen. These preclinical findings support further translational evaluation of this phage cocktail, including formal safety, pharmacokinetic, and dose-optimization studies before clinical testing.

In addition, we observed synergy between the phage cocktail and antibiotics such as PMB and MEM, which is consistent with phage-antibiotic synergy (PAS) (**Supplementary Figure 7**). These results suggest that the phage cocktail could be used in combination with existing antibiotic therapies. This is consistent with current clinical consensus, which recommends phage therapy as a complement, rather than a replacement, for standard antimicrobial treatment in difficult-to-treat infections (Alliance, Biotechnology, and Microbiology, 2023; Law et al., 2019).

In addition to the preclinical findings presented here, we are currently exploring the feasibility of this nebulized phage cocktail in an early clinical cohort. However, the *in vivo* therapeutic efficacy was evaluated using only a single clinical isolate (WK159), and further validation with a broader panel of MDR *P. aeruginosa* strains is necessary to confirm the generalizability of these findings. As these efforts remain preliminary, further studies are required to evaluate its safety, tolerability, and therapeutic potential in patients with refractory pulmonary infections. This will help determine whether the effects observed in experimental models can be translated into clinical benefit. Our study combines a targeted antimicrobial strategy based on a phage cocktail with a delivery approach suited to respiratory infections. These findings provide preclinical support for further investigation of nebulized phage cocktails and may help inform the future optimization of phage selection, formulation, delivery, and dosing strategies. Additional studies using clinically relevant infection models, pharmacokinetic and pharmacodynamic analyses, and controlled translational evaluation are needed before clinical application can be considered.

## 5 Conclusion

In this study, we developed a seven-phage cocktail for nebulized delivery targeting MDR *P. aeruginosa* pulmonary infections. The cocktail combined phages targeting both LPS and T4P receptors and showed broad antibacterial activity, biofilm-disrupting capacity, and activity in combination with conventional antibiotics. In a murine pneumonia model, high-dose nebulized administration improved survival, reduced bacterial burden, and alleviated tissue damage, while acute and long-term evaluations indicated favorable tolerability. These findings provide preclinical evidence supporting further investigation of nebulized phage cocktails as a potential therapeutic strategy for MDR *P. aeruginosa* respiratory infections.

## Data Availability

The datasets presented in this study can be found in online repositories. The names of the repository/repositories and accession number(s) can be found below: http://db.cngb.org/cnsa/project/CNP0009712_ee731f94/reviewlink/, CNP0009712.
