# Supplementary material for: A broad-host-range inhalable phage cocktail for treatment of multidrug-resistant Pseudomonas aeruginosa pulmonary infections
Source: Front Cell Infect Microbiol. 2026 Sep 15;16:1912918. doi: 10.3389/fcimb.2026.1912918 (PMC13619946; doi:10.3389/fcimb.2026.1912918)
Supplement: Supplementary file 1 [file DataSheet1.pdf]

# *Supplementary Material*

## 1 Supplementary Figures and Tables

### 1.1 Supplementary Figures

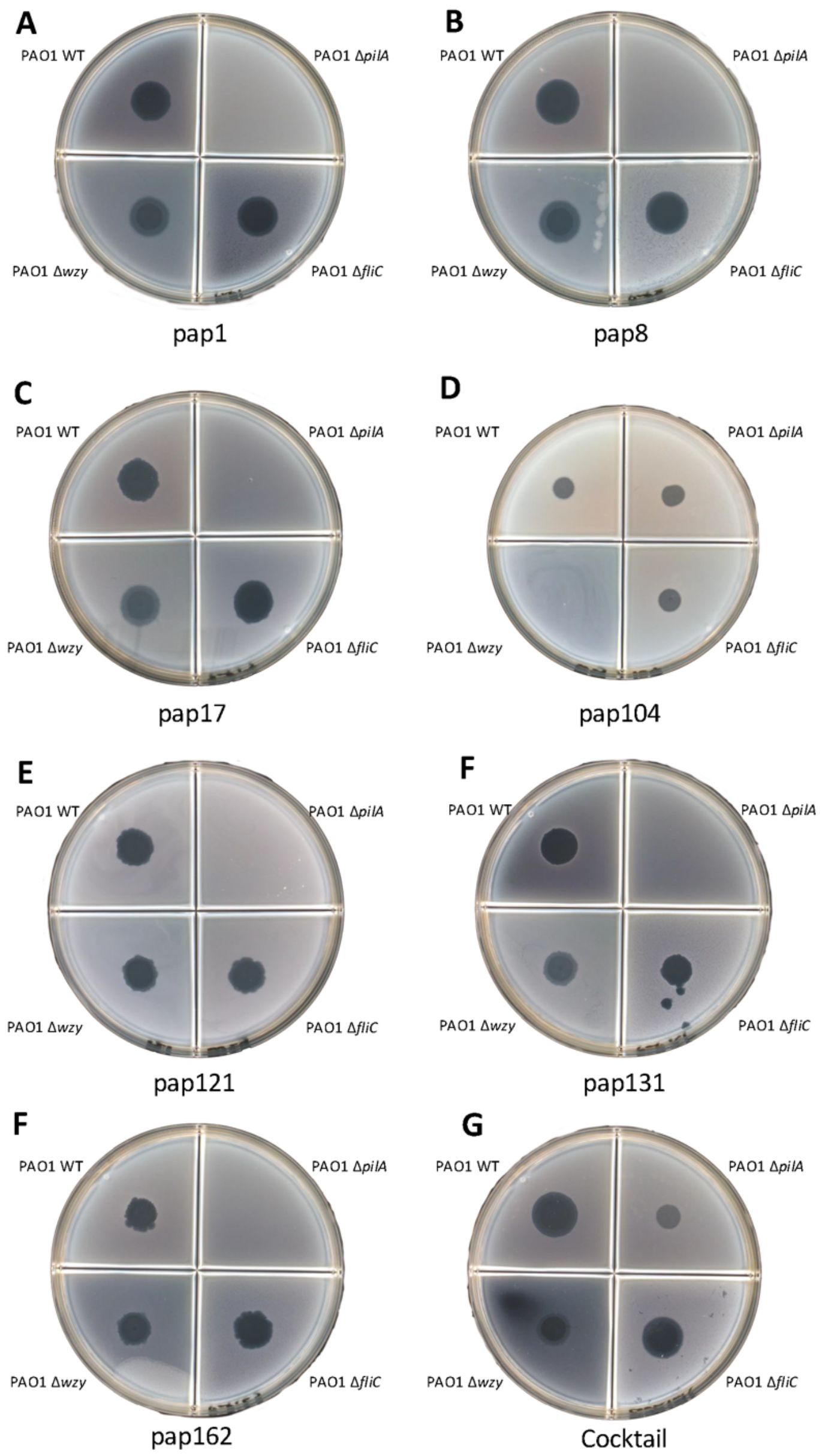

**Supplementary Figure 1. Susceptibility profiles of *P. aeruginosa* PAO1 wild-type (WT) and receptor-related gene mutant strains to different phages.** Each four-sector plate was inoculated with one of four strains: PAO1 WT, PAO1 Δ*pilA* (type IV pilus-deficient strain), PAO1 Δ*wzy* (LPS O-antigen-deficient strain), and PAO1 Δ*fliC* (flagellin-deficient strain). Phage suspensions (pap1, pap8, pap17, pap104, pap121, pap131, pap162, or a phage cocktail) were spotted onto the surface of the bacterial lawn. Clear plaques (lysis) were observed for the WT and specific mutant strains depending on the phage receptor, while the absence of the corresponding receptor (e.g., in Δ*pilA* or Δ*wzy* strains) resulted in resistance to certain phages, confirming receptor specificity.

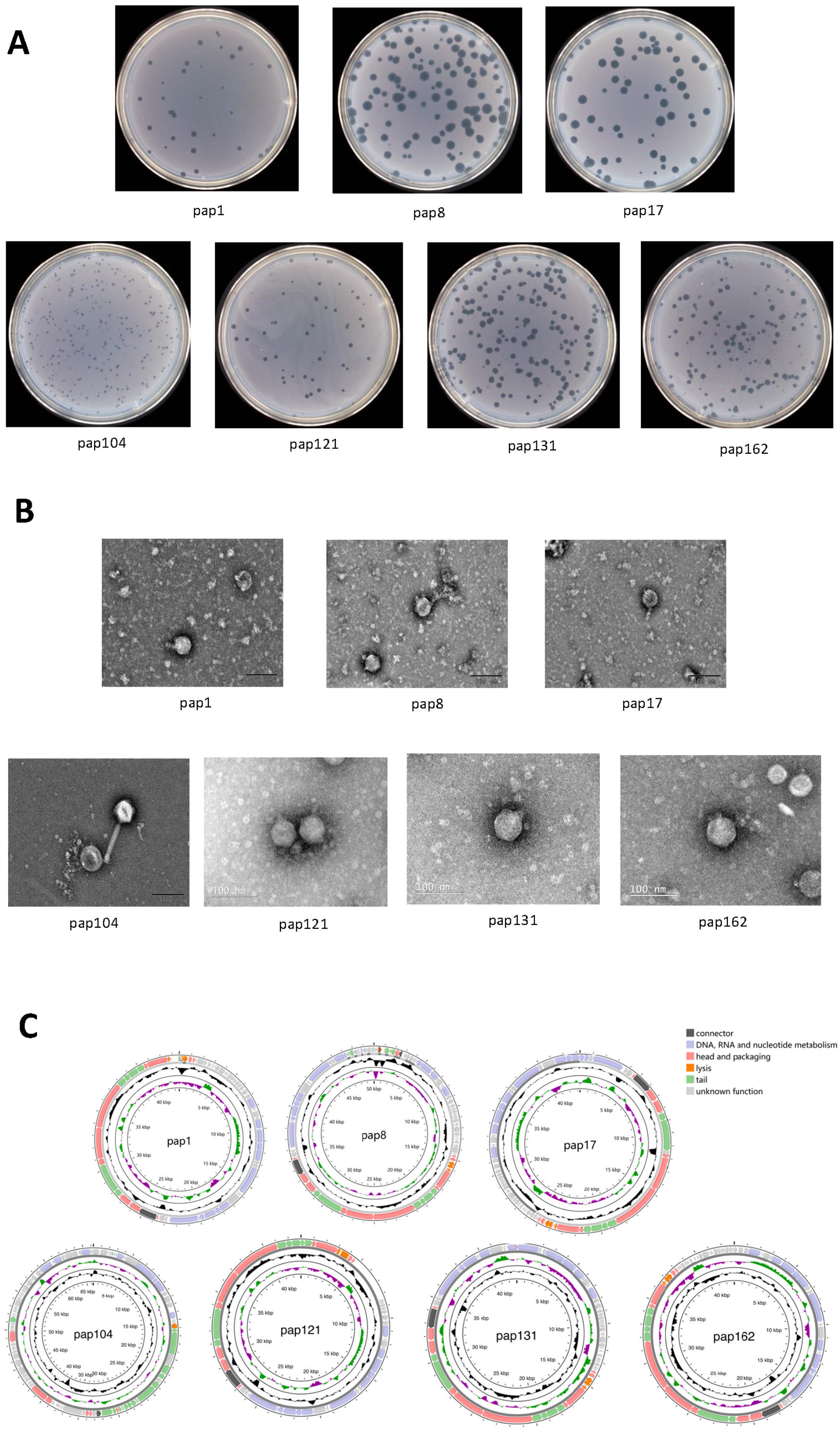
A
pap1
pap8
pap17
pap104
pap121
pap131
pap162
B
pap1
pap8
pap17
pap104
pap121
pap131
pap162
100 nm
C
connector
DNA, RNA and nucleotide metabolism
head and packaging
lysis
tail
unknown function
pap1
pap8
pap17
pap104
pap121
pap131
pap162

**Supplementary Figure 2. Phenotypic and genomic characteristics of the candidate phages.** (A) Plaque morphology of the phages on *P. aeruginosa* PAO1 host lawns. (B) Transmission electron microscopy images of phage particles. Scale bar: 100 nm. (C) Genomic organization and functional annotation of phage genomes. The linearized genome maps illustrate conserved functional modules, including those involved in connector, DNA, RNA and nucleotide metabolism, head and packaging, lysis, tail and unknown function. Phages are labeled as indicated: pap1, pap8, pap17, pap104, pap121, pap131, and pap162.

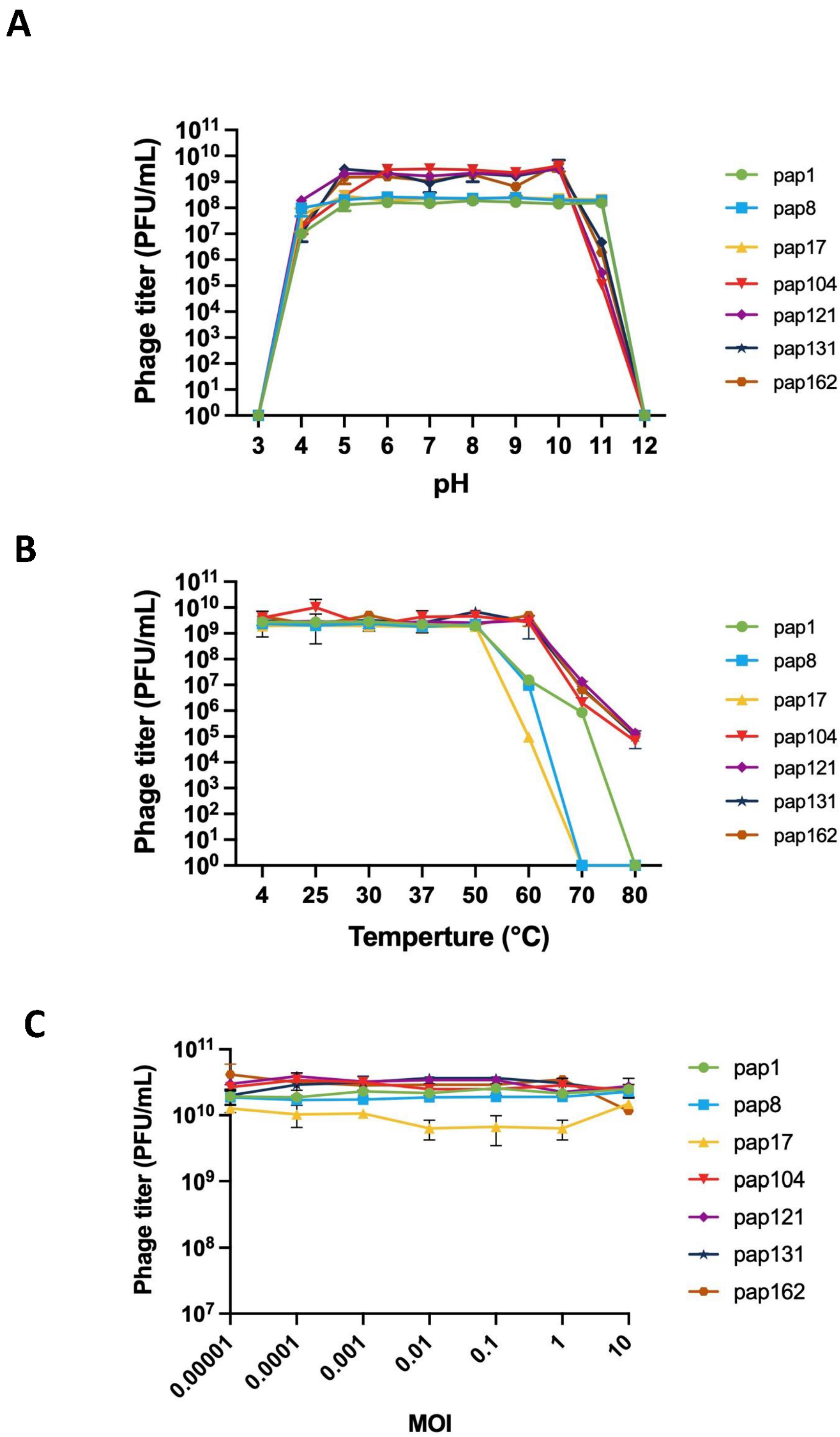
A
Phage titer (PFU/mL)
pH
pap1
pap8
pap17
pap104
pap121
pap131
pap162
B
Phage titer (PFU/mL)
Temperture (°C)
pap1
pap8
pap17
pap104
pap121
pap131
pap162
C
Phage titer (PFU/mL)
MOI
pap1
pap8
pap17
pap104
pap121
pap131
pap162

**Supplementary Figure 3. Biological characteristics of the candidate phages.** (A) pH stability of phages. Viral titers were determined across pH values ranging from 3 to 12. (B) Temperature stability of phages. Titers of the indicated phages were measured at temperatures from 4 °C to 80 °C. (C) Determination of optimal MOI of phages. Phage titer of each phage was evaluated at MOI values ranging from 0.00001 to 10. Error bars indicate replicate variability.

**A**

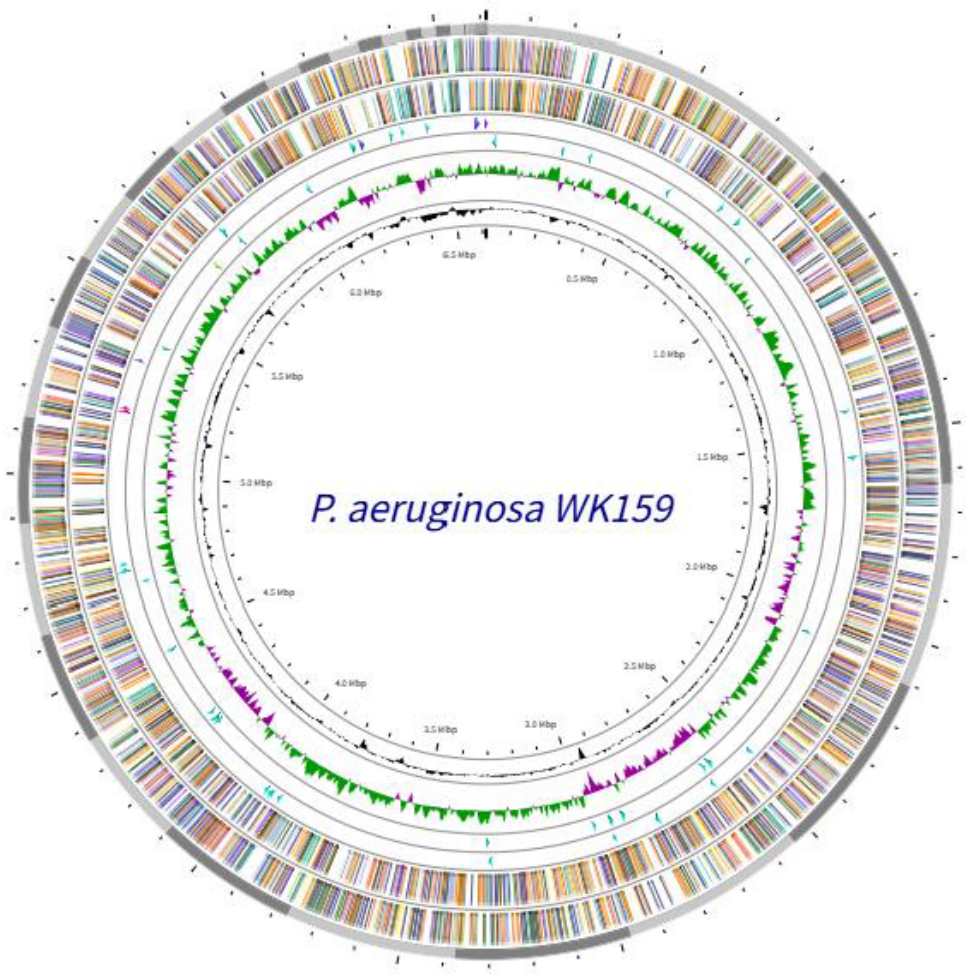


**Non-coding RNA categories**

- rRNA
- tRNA
- Repeat region
- tmRNA

**COG functional categories**

**INFORMATION STORAGE AND PROCESSING**

- Translation, ribosomal structure and biogenesis
- RNA processing and modification
- Transcription
- Replication, recombination and repair
- Chromatin structure and dynamics

**CELLULAR PROCESSES AND SIGNALING**

- Cell cycle control, cell division, chromosome partitioning
- Defense mechanisms
- Signal transduction mechanisms
- Cell wall/membrane/envelope biogenesis
- Cell motility
- Intracellular trafficking, secretion, and vesicular transport
- Posttranslational modification, protein turnover, chaperone

**METABOLISM**

- Energy production and conversion
- Carbohydrate transport and metabolism
- Amino acid transport and metabolism
- Nucleotide transport and metabolism
- Coenzyme transport and metabolism
- Lipid transport and metabolism
- Inorganic ion transport and metabolism
- Secondary metabolites biosynthesis, transport and catabolism

**POORLY CHARACTERIZED**

- Function unknown

**Supplementary Figure 4. Genetic structure characteristics of *P. aeruginosa* WK159.** The circular diagram presents the genome annotation profile of *P. aeruginosa* WK159. The multi-ring structure displays the genomic features of this strain from the outermost to the innermost circle: coding sequences on the forward strand, coding sequences on the reverse strand, non-coding RNA elements (tRNA and rRNA), and GC content. Functional annotations are color-coded based on COG database, with different colors representing distinct functional categories.

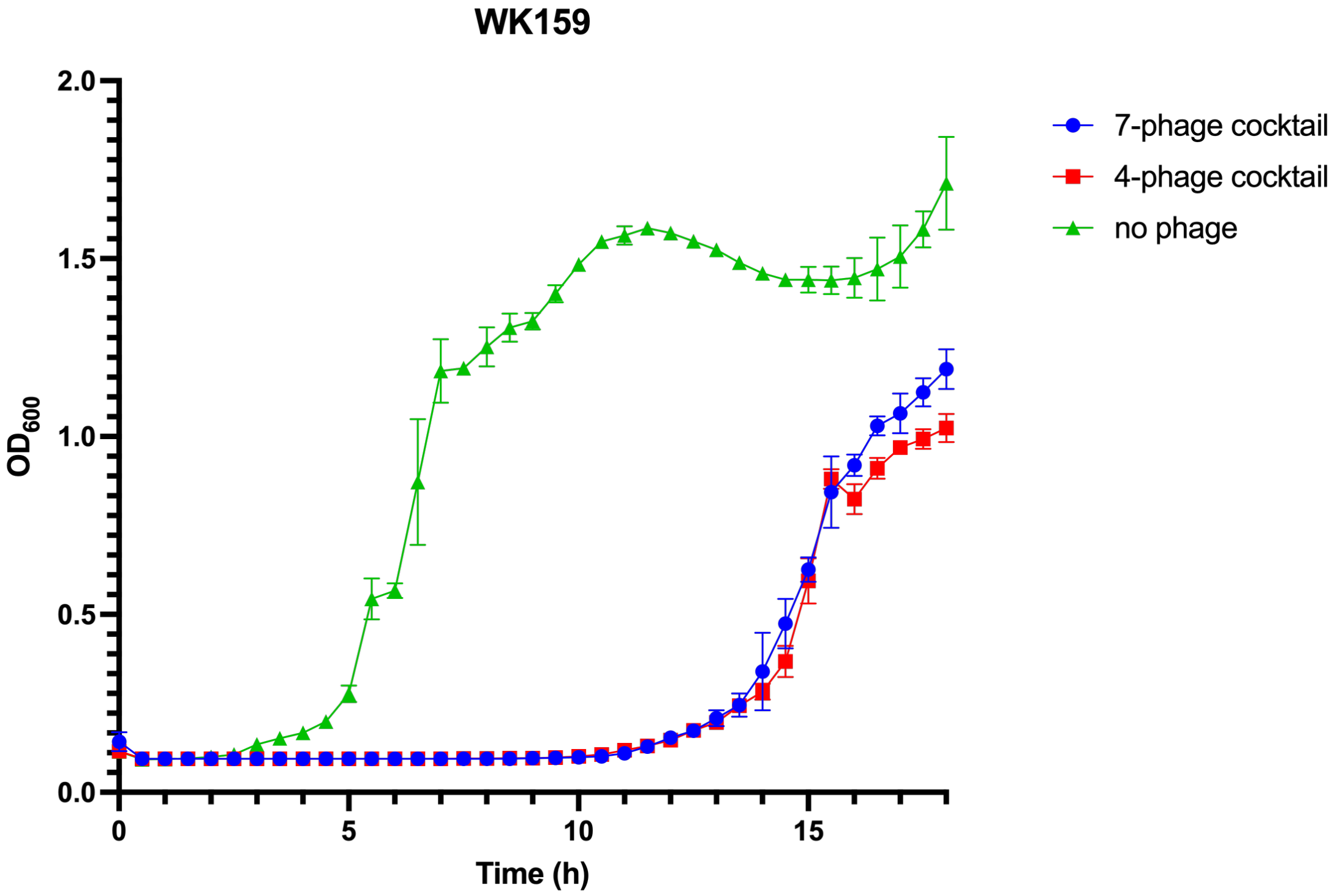


**Supplementary Figure 5. Comparison of the antibacterial activity of the complete seven-phage cocktail and the four-phage sub-cocktail against *P. aeruginosa* WK159.** Growth kinetics of *P. aeruginosa* WK159 treated with the complete seven-phage cocktail, the four-phage sub-cocktail (pap8, pap121, pap131, and pap162), or the no-phage control. Both cocktails were tested at a total MOI of 0.1, and $OD_{600}$ was monitored for 18 h. Data are presented as the mean ± SD (n = 3).

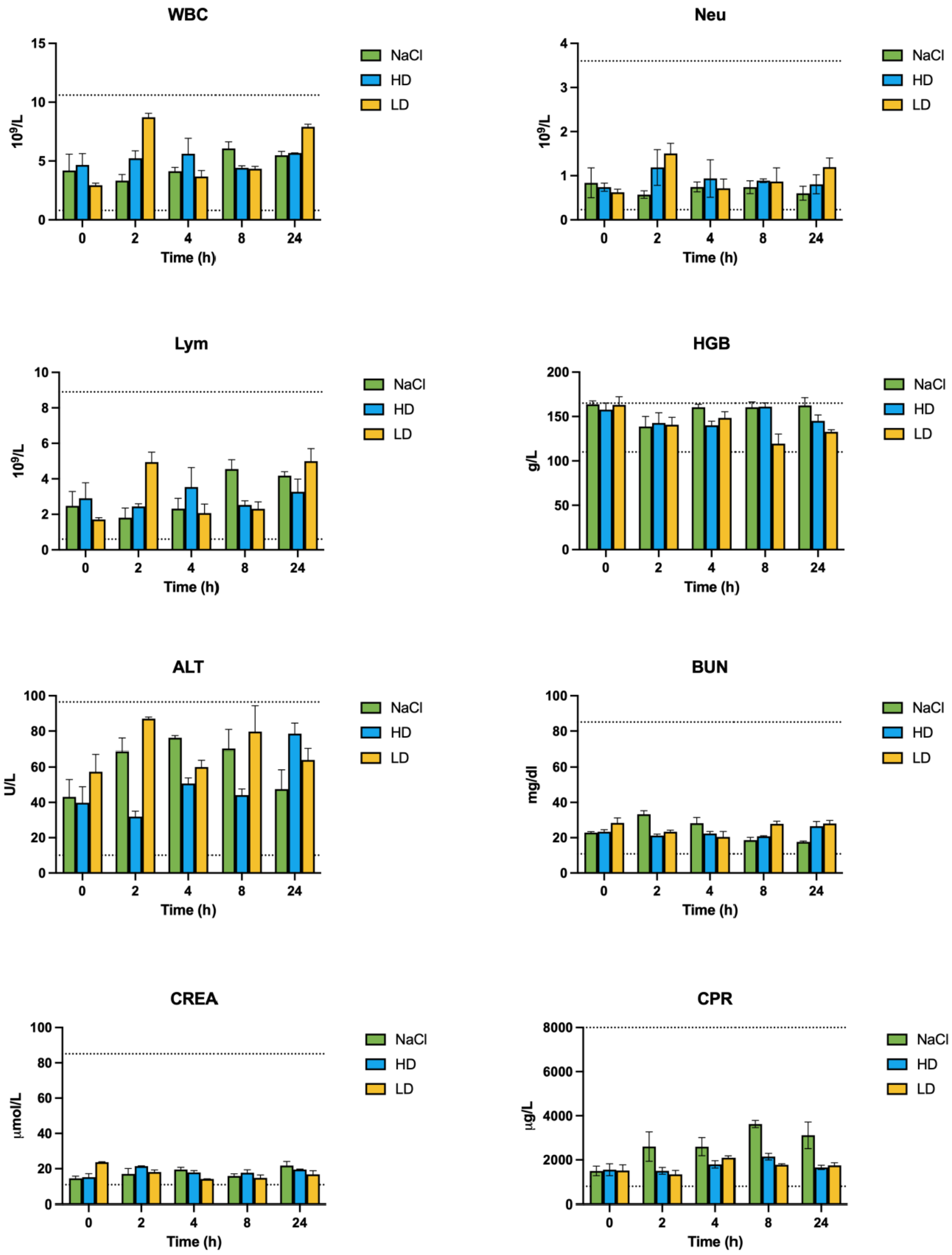
WBC
Neu
Lym
HGB
ALT
BUN
CREA
CPR
NaCl
HD
LD
10⁹/L
g/L
U/L
mg/dl
µmol/L
µg/L
Time (h)
0
2
4
8
24

**Supplementary Figure 6. Dynamic changes in hematological and biochemical parameters following single-dose nebulization exposure in mice.** The figure illustrates the profiles of peripheral blood and serum indices measured before administration (0 h) and at 2, 4, 8, and 24 h post-treatment. The assessed parameters include white blood cell count (WBC, $10^9$/L), neutrophil count (Neu, $10^9$/L), lymphocyte count (Lym, $10^9$/L), hemoglobin (HGB, g/L), alanine aminotransferase (ALT, U/L), blood urea nitrogen (BUN, mg/dL), creatinine (CREA, µmol/L), and C-reactive protein (CRP, µg/L). Data are presented for the NaCl control group (green), Cocktail HD (blue), and Cocktail LD (yellow). Dashed horizontal lines indicate established physiological reference ranges for each parameter. Data are presented as mean ± SD.

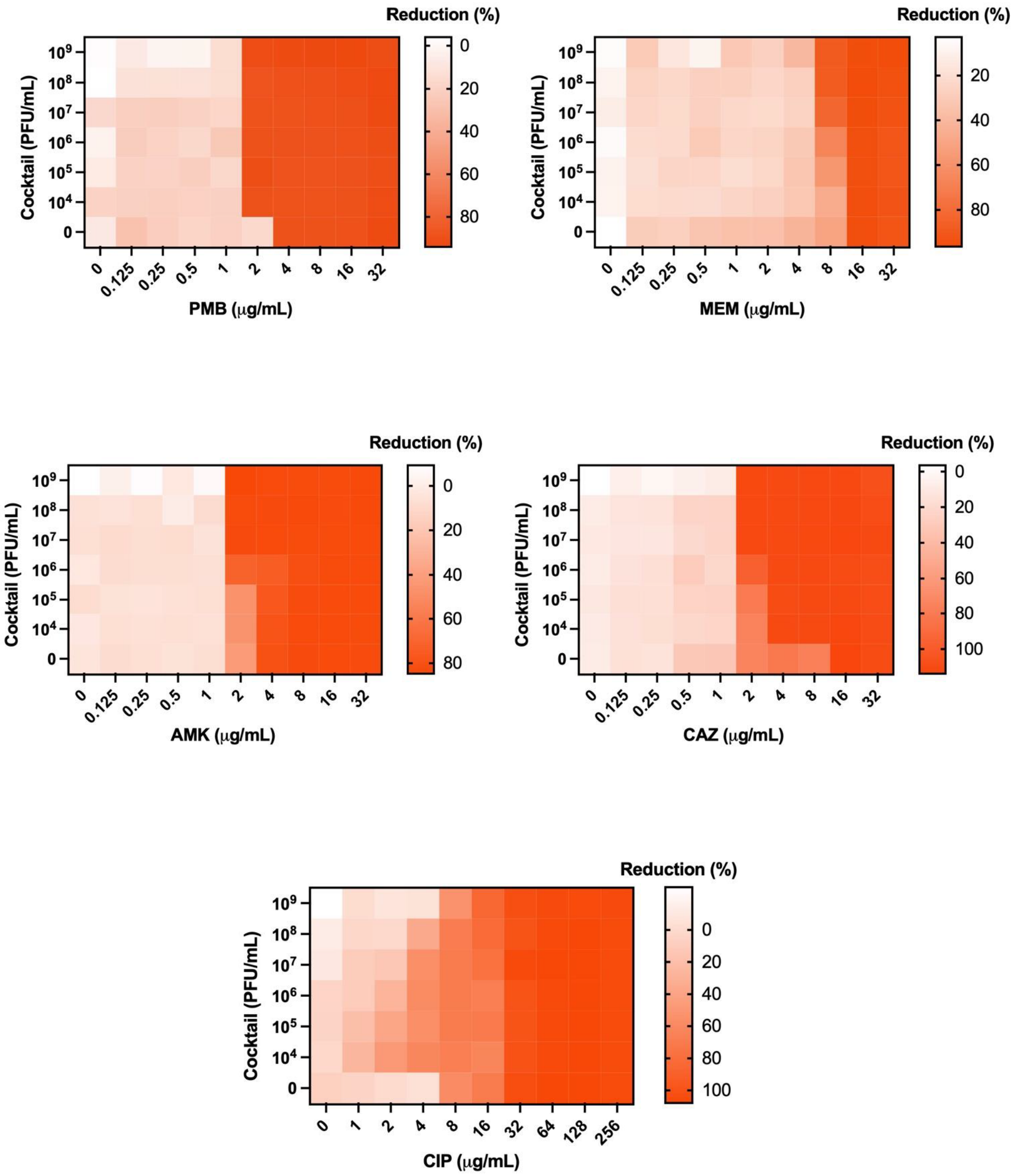
Reduction (%)
Cocktail (PFU/mL)
PMB (μg/mL)
MEM (μg/mL)
AMK (μg/mL)
CAZ (μg/mL)
CIP (μg/mL)
0
0.125
0.25
0.5
1
2
4
8
16
32
64
128
256
10⁹
10⁸
10⁷
10⁶
10⁵
10⁴
20
40
60
80
100

**Supplementary Figure 7. Heatmap of the synergistic effects between phage cocktail and antibiotics on *P. aeruginosa* WK159.** Each subplot represents one of five antibiotics tested: polymyxin B (PMB), meropenem (MEM), amikacin (AMK), ceftazidime (CAZ), and ciprofloxacin (CIP). The x-axis shows twofold serial dilutions of antibiotics (PMB, MEM, AMK, CAZ: 0.125-32 μg/mL; CIP: 1-256 μg/mL). The y-axis indicates phage titers ranging from $10^4$ to $10^9$ PFU/mL. Color intensity reflects the percentage of bacterial reduction, with darker shades representing greater reduction.

## 1.2 Supplementary Tables

**Supplementary Table 1. Clinical origins of *P. aeruginosa* isolates used for biofilm assays.**

| Strain No. | Clinical Diagnosis | Specimen Source |
|---|---|---|
| WK10 | Pneumonia, traumatic brain injury | Sputum |
| WK63 | Pneumonia, tracheotomy | Bronchoalveolar lavage fluid |
| WK121 | Pneumonia, traumatic brain injury | Sputum |
| WK159 | Pneumonia, paralysis | Sputum |
| WK221 | Pneumonia, diabetes mellitus | Deep venous catheter |
| WK257 | Pneumonia, traumatic brain injury | Sputum |

**Supplementary Table 2. Comprehensive resistance profile and corresponding resistance genotypes of multidrug- resistant clinical isolate WK159**

| Antimicrobial Category | Antimicrobial Agent | Phenotypic Result (S/I/R) | Phenotypic Result (MIC, µg/mL) | Zone Diameter (mm) | Resistant Gene(s) Detected | Genotype-Phenotype Concordance |
|---|---|---|---|---|---|---|
| Aminoglycoside | Amikacin | S | 4 | | aph(3')-IIb, aac(6')-Ib4 | NO |
| | Gentamycin | R | ≥16 | | aadA2 | YES |
| | Tobramycin | R | ≥16 | | aph(3')-Ia | YES |
| β-lactam/β-lactamase inhibitor combination | Ampicillin-Sulbactam | R | ≥32 | | blaPDC-142, blaOXA-395 | YES |
| | Cefoperazone/Sulbactam | S | | 22 | blaPDC-142, blaOXA-395 | NO |
| | Piperacillin-Tazobactam | I | 64 | | blaPDC-142, blaOXA-395 | YES |
| Penicillin | Piperacillin | I | | 24 | blaPDC-142, blaOXA-395 | YES |

| | | | | | |
|---|---|---|---|---|---|
| | Ampicillin | R | ≥32 | blaPDC-142, blaOXA-395 | YES |
| Cephalosporin (1st generation) | Cefazolin | R | ≥64 | blaPDC-142, blaOXA-395 | YES |
| Cephalosporin (2nd generation) | Cefotetan | R | ≥64 | blaPDC-142, blaOXA-395 | YES |
| Cephalosporin (3rd generation) | Ceftazidime | I | 16 | blaPDC-142, blaOXA-395 | YES |
| | Ceftriaxone | R | ≥64 | blaPDC-142, blaOXA-395 | YES |
| Cephalosporin (4th generation) | Cefepime | S | 8 | blaPDC-142, blaOXA-395 | NO |
| Carbapenem | Imipenem | R | ≥16 | blaPDC-142, blaOXA-395 | YES |
| Fluoroquinolone | Ciprofloxacin | R | ≥4 | Not Detected | YES |
| | Levofloxacin | R | ≥8 | Not Detected | YES |
| Monobactam | Aztreonam | I | 16 | blaPDC-142 | YES |

| | | | | | | |
|---|---|---|---|---|---|---|
| Nitrofuran | Nitrofurantoin | R | ≥512 | | Not Detected | YES |
| Polymyxin | Polymyxin B | S | | 18 | fosA-354827590 | NO |
| Sulfonamide combination | Trimethoprim-Sulfamethoxazole | R | ≥320 | | dfrA1, sul1 | YES |

*Susceptibility was interpreted according to the Clinical and Laboratory Standards Institute (CLSI M100) guidelines. S, susceptible; I, intermediate; R, resistant

**Supplementary Table 3. Comparison of the seven-phage cocktail titers before and after nebulization.**

| Experiment | Mean before nebulization (PFU/mL) | Mean after nebulization (PFU/mL) | $Log_{10}$ titer loss |
|---|---|---|---|
| 1 | $2.722 \times 10^{10}$ | $2.139 \times 10^{10}$ | 0.105 |
| 2 | $3.639\times 10^{10}$ | $2.778 \times 10^{10}$ | 0.117 |
| 3 | $2.111\times 10^{10}$ | $1.250 \times 10^{10}$ | 0.228 |
